# Revealing chiral cell motility by 3D Riesz transform-differential interference contrast microscopy and computational kinematic analysis

**DOI:** 10.1038/s41467-017-02193-w

**Published:** 2017-12-19

**Authors:** Atsushi Tamada, Michihiro Igarashi

**Affiliations:** 1 Center for Transdisciplinary Research, Institute for Research Promotion, Niigata University, Niigata, 951-8510 Japan; 2 Department of Neurochemistry and Molecular Cell Biology, Graduate School of Medical and Dental Sciences, Niigata University, Niigata, 951-8510 Japan; 3 Decoding and Controlling Brain Information, Precursory Research for Embryonic Science and Technology, Japan Science and Technology Agency, Kawaguchi, Saitama, 332-0012 Japan

## Abstract

Left–right asymmetry is a fundamental feature of body plans, but its formation mechanisms and roles in functional lateralization remain unclear. Accumulating evidence suggests that left–right asymmetry originates in the cellular chirality. However, cell chirality has not yet been quantitatively investigated, mainly due to the absence of appropriate methods. Here we combine 3D Riesz transform-differential interference contrast (RT-DIC) microscopy and computational kinematic analysis to characterize chiral cellular morphology and motility. We reveal that filopodia of neuronal growth cones exhibit 3D left-helical motion with retraction and right-screw rotation. We next apply the methods to amoeba *Dictyostelium discoideum* and discover right-handed clockwise cell migration on a 2D substrate and right-screw rotation of subcellular protrusions along the radial axis in a 3D substrate. Thus, RT-DIC microscopy and the computational kinematic analysis are useful and versatile tools to reveal the mechanisms of left–right asymmetry formation and the emergence of lateralized functions.

## Abstract (web summary)

The lack of an appropriate method has hampered quantitative measurements of cell chirality. Here, the authors combine Riesz transform-differential interference contrast microscopy and computational kinematic analysis to reveal chiral cell motility of neuronal growth cone filopodia and cellular slime mold.

## Introduction

Bilateral biological organisms have the left–right axis that is specified with reference to the anterior-posterior and the dorsal-ventral axes. Most of the body structures form mirror images about the midline, but some of them are asymmetric along the left–right axis. Left–right asymmetry is a fundamental property that is widely observed across species, such as in the position of visceral organs and lateralized brain functions^1,2^. Despite a significant influence of left–right asymmetry on the body plan, its precise phenomenon, underlying molecular mechanisms and functional roles in the organisms still remain unclear^3^. With regard to the initial symmetry-breaking step, it was postulated that the molecular handedness or chirality is converted to a cellular and multicellular asymmetry that finally leads to left–right asymmetry in the organisms^4^. In accordance with this hypothesis, many recent reports demonstrated the existence of chirality at the cellular level^5–16^. Cell chirality is emerging as a key geometric property at the intermediate levels that may link the molecular chirality, mostly in cytoskeletons and motor proteins, to the left–right asymmetry at the higher levels^17,18^. However, to date, no systematic quantitative methods were available that could analyze the cell chirality that mostly appears in 3D space. Here we developed two essential techniques for visualizing and analyzing 3D cellular structures and motions, especially for studying the cell chirality.

Live imaging is an effective tool to visualize the cellular morphology and motility^19–21^. The first standard choice could be fluorescence imaging, but its application is practically limited due to the problem of phototoxicity^21,22^, which hampers 3D imaging of photosensitive fragile cellular structures with high-spatiotemporal resolutions. In the present study, we propose an alternative imaging technique that utilizes differential interference contrast (DIC) microscopy^21^. DIC microscopy, which creates contrast in unstained specimens with less phototoxicity, has been frequently used in 2D live cell imaging. However, due to the nonlinear shadow-cast image property along the shear axis of the prism, DIC microscopy has been considered to be unsuitable for 3D image reconstruction and intensity-based processing. To overcome this problem, many methods have been developed to date^23^. One of the most efficient and convenient methods adopts acquisition of multiple phase gradient images with orthogonal shears and their integration by the inverse Riesz transform (RT)^23–25^. RT^26^, which was independently and simultaneously proposed as the spiral phase transform^27^, is a multidimensional extension of the 1D Hilbert transform (HT), and has recently been used in many fields of image processing and analysis^28–31^. The inverse RT-based methods with multiple DIC images precisely restore original images, but they require special equipment and multi-shot image acquisition that is disadvantageous for fast 3D live imaging. A method for single-shot DIC imaging with HT was also developed^32^, but it cannot detect objects along the shear direction. Here we developed a simple but efficient method for single-shot DIC images with a composite Fourier filtering based on the directional RT^28^. This composite RT, utilizing both phase gradient and absorption information of DIC images, converts a shadow-cast DIC image into a self-luminous intensity image. This improved DIC microscopy with the composite RT, called RT-DIC microscopy, was applied to 3D time-lapse imaging of photosensitive structures.

In the step of “analysis”, information on the morphology and the motility should be extracted from the acquired images. The image analysis has long been performed by intuitive human vision. However, reproducible and standardized protocols are required for multidimensional large-scale image data that are beyond human perception^33,34^. In this study, we established computational methods to analyze voxel-wise kinematics of 3D cell motility, by incorporating the techniques of the structure tensor^35^ and the optical flow^36,37^ that were developed in the field of computer vision.

RT-DIC microscopy and the associated computational methods were then applied to detect chiral cellular morphology and motility. We first applied them to the motility analysis of the neuronal growth cone at the tip of extending neurites^38,39^. We previously reported that the needle-like filopodia extending from a growth cone body rotate in the direction of a right-handed screw by a conventional 2D DIC imaging and manual tracking of the filopodia on a single 2D focal plane^6^. This filopodial rotation drives asymmetric clockwise turning of neurites^5^. However, 2D imaging and analysis could not reveal the precise nature of the growth cone motility that occurs in 3D environments. By applying the newly developed methods, we succeeded in full visualization of 3D growth cone motility. The computational analysis uncovered a helical motion of growth cone filopodia with overall retraction and right-screw rotation.

We further applied these methods to analyze the cellular slime mold *Dictyostelium discoideum*, which is a unicellular organism that has been used as a model for studying cell migration, cell differentiation, and morphogenesis of multicellular structures^40^. We newly found two types of cell chirality, right-handed clockwise cell migration on 2D substrate, and right-screw rotation of subcellular protrusions along the radial axis in 3D substrate. RT-DIC microscopy and the computational kinematic methods are useful and versatile tools for quantitative analysis of 3D cell morphology and motility, and in particular for detection of the cell chirality, as demonstrated in the discovery of chiral motilities in the neurons and the cellular slime mold.

## Results

### Composite RT converts DIC images into self-luminous images

**Fig. 1:**
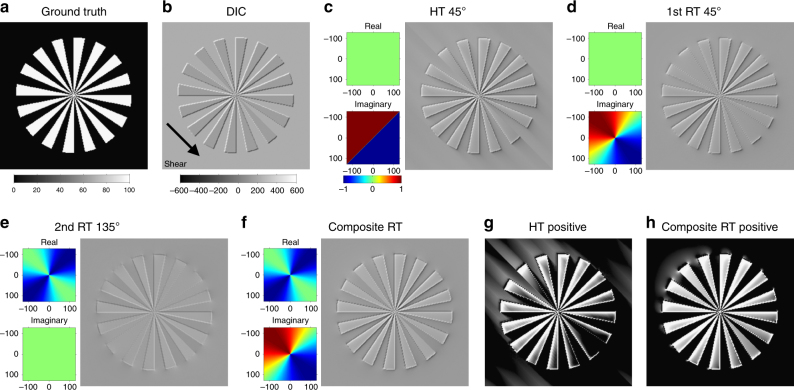
Conversion of a shadow-cast DIC image into a self-luminous image with RT. **a** Radial grating image. **b** DIC image synthesized from **a** by the sum of the inverted image and the gradient along the 45° shear axis. **c** HT of **b** with a 45°-directed 2D Fourier filter (left panels). Note the absence of features but generation of line artifacts along the shear axis. **d** RT of **b** with a 45° RT filter (left panels) selectively extracts 135° features. **e** 135°-oriented second-order RT of **b** extracts and inverts 45° features. **f** Composite of the first- and second-order RT detects features in all directions. **g**, **h** Thresholding of **c**, **f** by positive values. The composite RT **h** efficiently restores the ground truth image **a** without generating large artifacts

RT^26,27^ is the multidimensional extension of Hilbert transform (HT), which shifts the phase of 1D signals by 90° (Supplementary Note 1). We utilized a directional form of RT^28^. To test the effectiveness of RT-based image conversion, a ground truth radial grating image (Fig. 1a) was inverted and differentiated along the oblique shear axis to generate a synthetic DIC image consisting of both phase and absorption information (Fig. 1b). As previously reported^32,41^, HT directed along the shear axis effectively removed the shadows from the DIC image (Fig. 1c). However, HT did not allow visualization of the features oriented along the shear axis, but generated artifactual shadows in the surrounding region. Because the artifacts were likely to be caused by an abrupt change in the HT frequency filter, we tested a smoother RT filter directed to the shear axis. As we expected, RT restored the features with narrower orientation selectivity than HT, but did not generate artifacts with long shadows (Fig. 1d). To utilize the absorption information, we constructed another orthogonally oriented filter for 180° phase-shifting with a second-order RT. This filter selectively passed and inverted the features oriented along the shear axis (Fig. 1e). We constructed a composite filter of the first-order RT directed to the shear axis and the second-order RT oriented along the orthogonal axis. This filter removed the shadows and restored the features aligned in all orientations without generating large artifacts (Fig. 1f). Thresholding of the transformed images demonstrated that the composite RT restored the ground truth image much more efficiently than HT (Fig. 1g, h). The composite RT for a single-shot image was also compared with the inverse RT^23–25^ for multi-shot images that require acquisitions with 2–4 biases for each orthogonal shear direction. We found that the composite RT restores the ground truth object but generates a less isotropic image with diffuse artifacts than the inverse RT (Supplementary Fig. 1). We further demonstrated that such unwanted properties can be largely removed by appropriate filter settings and that the conversion efficiency can be raised enough for practical use.

### RT-DIC microscopy visualizes fine cellular structures

**Fig. 2:**
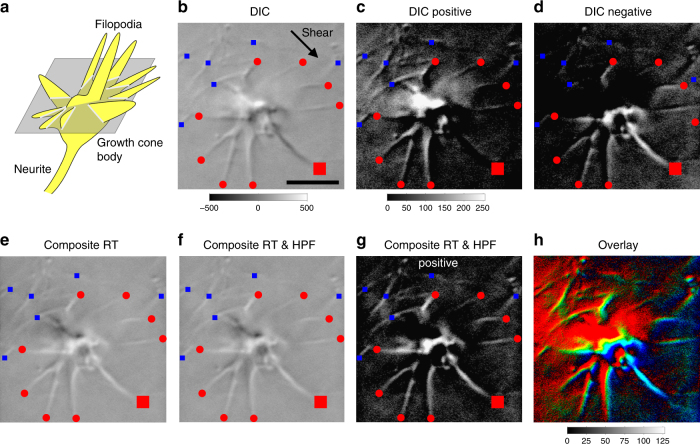
Composite RT-based conversion of a growth cone DIC microscopic image. **a** Schematic of a growth cone growing upward (yellow) and its optical section (light gray) in a focal plane (gray) with a DIC microscope. **b** DIC microscopic image capturing the frontal view of a neuronal growth cone embedded in collagen gels. Radially extending filopodia (red circles and a red square) and collagen fibers (blue squares) are visible. **c** Thresholding of **b** by positive values. **d** Thresholding of **b**, showing negative intensity only. **e**, **f** The composite RT of **b** without **e** or with **f** high-pass filtering. All fiber structures including a 45°-oriented dark filopodium (red square) are converted to bright objects without shadows. Note that shading bias in the upper-left quadrant in **e** is removed in **f**. **g** Self-luminous image obtained by thresholding of **f**. **h** Overlay of **c** (red), **d** (blue) and **g** (green). Data from cell #2. Scale bars, 5 μm

The composite RT was applied to real DIC microscopic images (Fig. 2). A test image was acquired from a growth cone of a neuron that was cultured within 3D collagen gels (Fig. 2a). The growth cone body in the center radially extended multiple filopodia. DIC microscopy gave an optically sectioned image capturing fine structures of the growth cone with filopodial processes (Fig. 2b). However, the shadow-cast appearance along the shear axis made the image difficult to interpret quantitatively. Simple thresholding at zero intensity extracted only partial features representing the positive or negative slope of the gradient (Fig. 2c, d). After testing various filters based on HT and RT (Supplementary Fig. 2), we found that the composite RT (Fig. 1f) effectively removed the shadows from the DIC image and restored the full features of the growth cone (Fig. 2e). In addition, we found that the inevitable background shading in DIC microscopy^21^, which was evident in the upper-left quadrant (Fig. 2b–e), can be corrected by cutting low-frequency components with a high-pass filter (Fig. 2f, Supplementary Note 1). Thresholding of this image by positive values enabled extraction of a fluorescence-like self-luminous intensity image (Fig. 2g). An overlay of the source DIC and RT-filtered DIC images demonstrated that DIC detected the positive and negative slopes of the object, whereas RT-filtered DIC detected its central ridge (Fig. 2h). We call this DIC microscopic technique that was improved with the composite RT filtering “RT-DIC microscopy”. An ImageJ plugin (Supplementary Software 1) and MATLAB codes (Supplementary Software 2) for RT-DIC conversion are available.

### RT-DIC microscopy enables 3D imaging of photosensitive cells

**Fig. 3:**
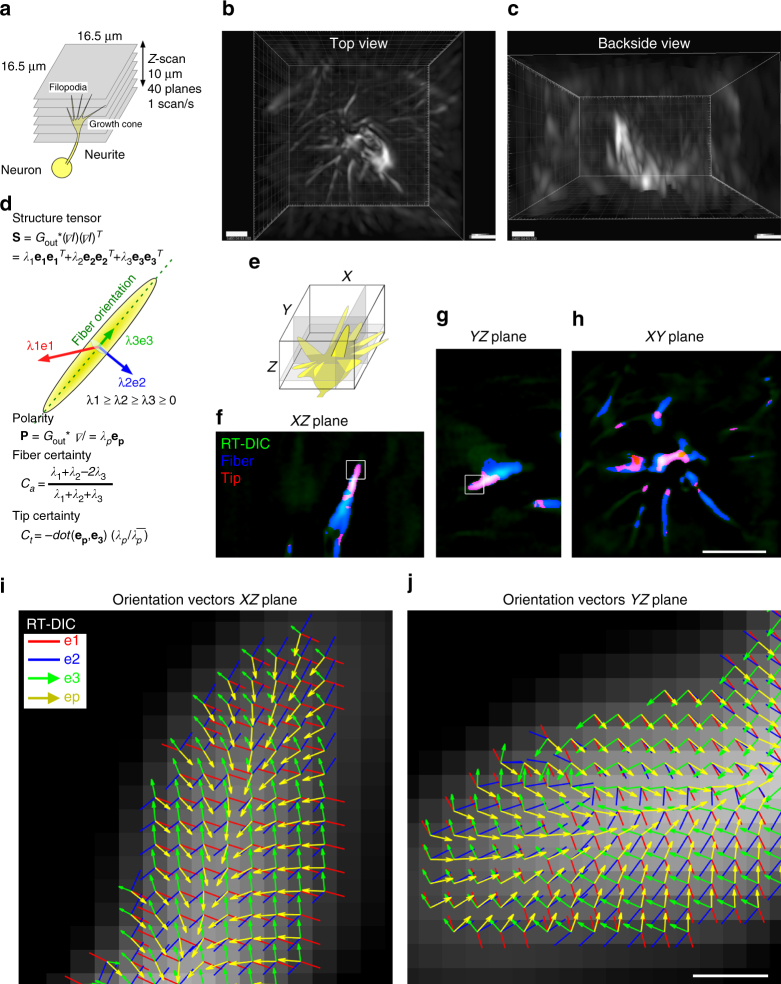
3D RT-DIC time-lapse imaging and morphological analysis of the growth cone. **a** Set-up for acquisition of time-lapse and Z-scanned DIC images. **b**, **c** Top **b** and backside **c** views of rendered RT-DIC images. **d** Voxel-wise geometric feature estimation with the structure tensor. Structure tensor **S**, which is calculated from the cross product of the gradient of intensity *I* and smoothing with a Gaussian *Gout*, is eigen decomposed. Eigenvector **e3** with the lowest eigenvalue *λ3* represents the fiber orientation. Polarity vector **P**, which is calculated from the smoothed gradient, is represented by the magnitude *λp* and the orientation **ep**. Fiber certainty *Ca* is given by the eigenvalues. Tip certainty *Ct* is given by **ep**, **e3**, *λp* and its mean . **e** A schematic representing cross sectioning of a 3D RT-DIC image stack with *XZ*, *YZ*, and *XY* planes (gray). **f**–**h** Cross-section images of RT-DIC intensity (green) were overlaid with fiber (blue) and tip (red) indices. **i**, **j** Distribution of the tensor-derived eigenvectors and the polarity vector at the filopodial tip (boxed regions in **f** and **g**). Data from cell #2. Scale bars, 2 μm at lower left corners of **b** and **c**, 5 μm in **h** for **f**–**h**, 200 nm in **j** for **i** and **j**

RT-DIC microscopy was applied to 3D time-lapse imaging of the growth cones, photosensitive and motile structures that could not be visualized by standard fluorescence microscopy, due to the problem of the phototoxicity especially in case of highly frequent sampling (Supplementary Fig. 3, Supplementary Movie 1). Optically sectioned DIC image stacks of a growth cone that was growing upward within collagen gels were acquired using an upright DIC microscope equipped with a fast piezo motor-driven focusing system (Fig. 3a, Supplementary Movie 2). 3D RT-DIC images were further post-processed for 3D deconvolution, spatiotemporal smoothing and normalization of intensity and voxel size (Supplementary Fig. 4). Multiple needle-like filopodia elongated upward from the growth cone body beneath the volume of interest (Fig. 3b, c). Time-lapse movies visualized precise 3D morphology and motility of growth cone filopodia, which had not been previously visualized with conventional 2D imaging^6^ (Supplementary Movie 3).

### Structure tensor decomposes complex 3D cell morphology

Successful 3D imaging and visualization with RT-DIC microscopy highlighted the need for tools to quantitatively analyze the morphology and the motility. When a cell has simple round-shaped morphology without large deformation, it can be represented as a sphere or an ellipsoid with a few parameters like the center of the mass or the diameter. In contrast, when a cell has complex morphology with ever-changing processes like the growth cone filopodia, the morphology cannot be represented with simple parameters. Furthermore, high dimensional data along 3D space and time cannot be analyzed manually with human visual perception. Here we attempted to develop automated, quantitative, and versatile methods for analyzing multidimensional image data along 3D space and time axes, by incorporating the notion developed in the field of computer vision^33^ (Supplementary Fig. 4).

For morphological analysis, we introduced the structure tensor, which is the second moment matrix of image intensity^35^ (Supplementary Note 2, Supplementary Software 3). The local geometric feature at each voxel can be estimated by eigen decomposition of the tensor (Fig. 3d). The voxel-wise local feature is represented by the weighted combination of the three basic shapes; sphere, fiber, and plane. The eigenvector with the least eigenvalues represents the orientation of fiber structures. Images can be segmented by thresholding with intensity, retrograde tractability with orientation vectors, and the certainty indices (Fig. 3d). These geometric feature analysis were applied to 3D RT-DIC images of the growth cone (Fig. 3e–j). We succeeded in extraction of fiber and tip features (Fig. 3e–h) and estimation of orientation vectors (Fig. 3i, j) on the cross-sections. Time-lapse movies (Supplementary Movie 4) visualized spatiotemporal distribution pattern of fiber and tip features.

### Optical flow analysis reveals helical motion of growth cone

For quantification of 3D motility, we developed automated and voxel-based methods for 3D kinematic analysis combined with the optical flow analysis^36,37^ (Supplementary Note 3, Supplementary Software 4). The validity of these methods was tested with a virtual helical motion of a sphere in 3D space (Supplementary Fig. 5a). Voxel-wise single-frame and double-frame velocity vectors between two image frames were estimated by an optical flow analysis^36^ modified for 3D images (Supplementary Fig. 5b). From these optical flow vectors, translational physical quantities including velocity, acceleration, jerk (Supplementary Fig. 5c) and rotational quantities including tangent, normal and binormal unit vectors and angular velocity (Supplementary Fig. 5d) were estimated for each voxel. These voxel-wise quantities were represented as vector fields with functions of space and time. These estimated quantities were well matched with the ground truth values (Supplementary Fig. 5e, Supplementary Table 1).

**Fig. 4:**
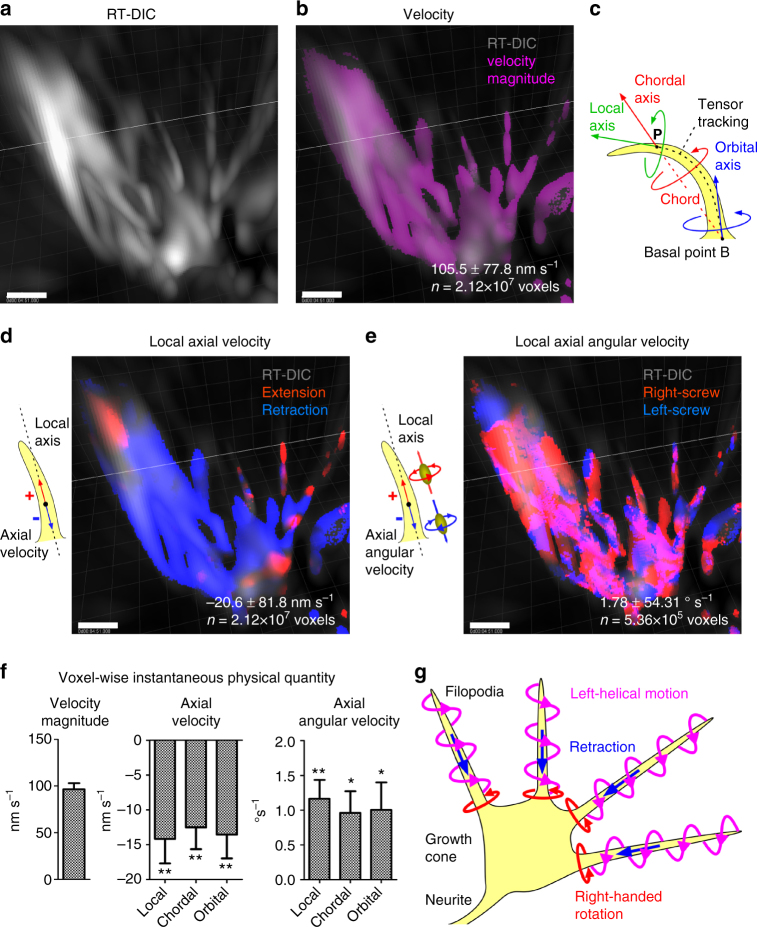
Kinematic analysis of growth cone motility. **a** RT-DIC intensity image from oblique view. Growth cone from cell #2. **b** Distribution of velocity magnitude (magenta) overlaid on the RT-DIC intensity (gray). **c** Definition of the local, chordal and orbital axes at the point of interest P. **d** Distribution of axial velocity along the local axis. Positive (red) and negative (blue) values represent extension and retraction, respectively. **e** Distribution of axial angular velocity along the local axis. Positive (red) and negative (blue) values represent right-screw and left-screw rotation, respectively. The values in **b**, **d**, and **e** represent the mean ± s.d. for all voxels in all frames in the images of this growth cone. Their histograms are shown in Supplementary Fig. 6. All of the individual data for eight growth cones are shown in Supplementary Tables 2, 3. **f** Summary of voxel-wise instantaneous physical quantities. The values represent mean ± s.e.m. of the means for individual cells (*n* = 8). *P < 0.05, **P < 0.01. One sample *t*-test for the null hypothesis that the mean is equal to zero. **g** Chiral helical motility of growth cone filopodia revealed by the kinematic analysis. Scale bars, 1 μm

We applied the kinematic methods described above to real 3D RT-DIC time-lapse images of the growth cone. We first calculated instantaneous and local 3D optical flow velocity vectors for each voxel in each frame (Supplementary Note 3). Overlay of the velocity magnitude on the RT-DIC intensity revealed that the calculated velocity was high at motile filopodia, consistent with visual perception (Fig. 4a, b, Supplementary Movie 5). Local axial velocity was calculated for visible and tractable voxels as a projection of the vector onto the local fiber direction that was estimated by the structure tensor analysis (Fig. 4c). Spatial distribution of the local axial velocity indicated that the trunk of filopodia tended to retract toward the growth cone body, whereas the tip shows both extension and retraction (Fig. 4d, Supplementary Movie 6). Angular velocity vectors were estimated from four velocity vectors derived from five sequential frames (Supplementary Note 3). Local axial angular velocity was calculated for visible, tractable, and motile voxels as a projection of the angular velocity vector onto the local fiber direction. Spatial distribution of the axial angular velocity indicated that the right-screw rotation was dominant compared with the left-screw rotation (Fig. 4e, Supplementary Movie 6). The histograms of instantaneous and local physical quantities indicated high motility of filopodia (Supplementary Fig. 6a), tendency of retraction along local axis (Supplementary Fig. 6b) and dominance of right-screw rotation about local axis (Supplementary Fig. 6c). Particle tracking analysis (Supplementary Movie 7) further visualized that individual filopodia not only twirled in the direction of a right-handed screw as previously reported^6^ but also exhibited axial spinning. They also showed retrograde motion toward the growth cone body, consistent with the retrograde actin flow^38^. We also calculated physical quantities along the chordal axis defined by the chord to the tracked basal point, and those along the orbital axis defined as the direction at the basal point (Fig. 4c). Individual (Supplementary Tables 2, 3) and summarized (Fig. 4f) statistical data indicate that velocity was negative and that the angular velocity was positive in all three types. The analyses revealed that growth cone filopodia tend to retract backward and rotate in the right-screw direction, showing a left-helical helical motion along their longitudinal axis (Fig. 4g).

### Quantification of chiral pattern in clockwise neurite growth

**Fig. 5:**
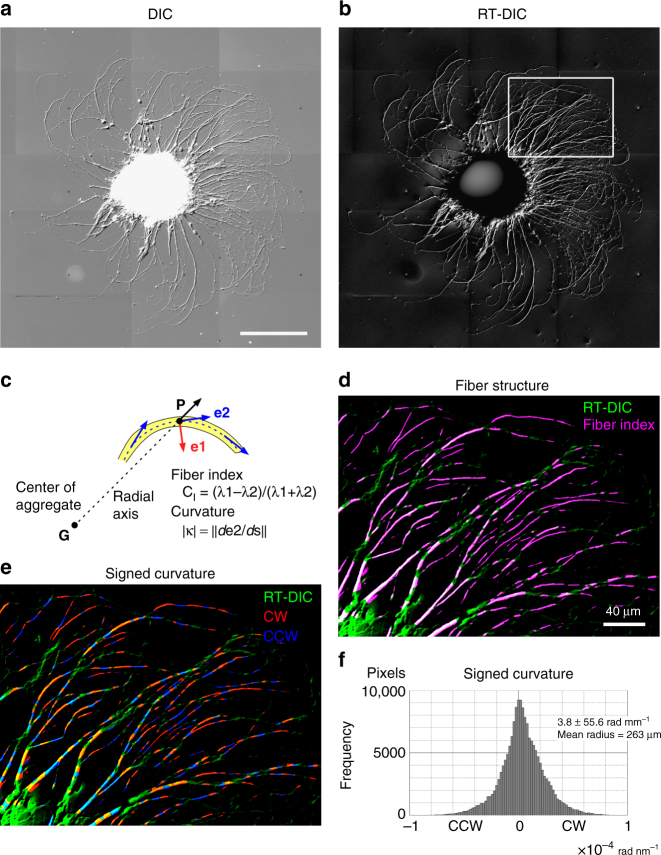
Quantification of chirality in the clockwise neurite growth on 2D surface. **a** A montaged still DIC image of a hippocampal neuronal re-aggregate cultured for 48 h. Neurites extended from the re-aggregate turned clockwise as previously reported^5,6^. **b** RT-DIC image of **a**. Neurites appear as self-luminous objects. **c** Feature estimation with the structure tensor. Neurites are extracted by the eigenvalue-derived fiber index. The curvature of a neurite is calculated as a differential of **e2** along the arc formed by **e2**. The curvature is signed by the radial axis. **d** Neurite fragments extracted by the fiber index. Only the rectangular region in **b** is shown. **e** Distribution of signed curvature on the extracted neurite fragments. Positive (red) and negative (blue) values represent clockwise (CW) and counterclockwise (CCW) rotation, respectively. **f** Histogram of the signed curvature. The values represent mean ± s.d. (*n* = 1.7 × 10^5^ pixels). *P* < 0.0001 by Wilcoxon signed-rank test against zero. Scale bars, 200 μm in **a** for **a** and **b**, 40 μm in **d** for **d** and **e**

RT-DIC microscopy and the associated methods can be used to automatically analyze chiral patterns in various scales, dimensions and cell types. In the scale larger than the growth cone, the neurons extend neurites clockwise on 2D culture substrates^5,6^ (Fig. 5a). This 2D clockwise neurite growth is driven by the 3D right-screw rotation of the growth cone filopodia^6^, which is newly found to be accompanied by the 3D helical motion as described in the previous section. Even for such simple chiral pattern in a single still 2D image, there has been no other way than to perform time-consuming manual tracking and measurement^6^. Here, the DIC image was converted to the RT-DIC intensity image (Fig. 5b), which was further processed for calculation of the fiber index and the curvature from the structure tensor (Fig. 5c). We succeeded in extraction of fiber fragments (Fig. 5d) and calculation of the signed curvature at every pixel on the neurite (Fig. 5e, f) within a few min. The curvature faithfully represents both local clockwise and counterclockwise neurite turning, with a clockwise bias. The calculated mean radius matches with visually inspected values. These results ensure a high degree of usability for the current methods in automatic quantification of chiral patterns.

### Cellular slime mold shows clockwise migration on 2D surface

**Fig. 6:**
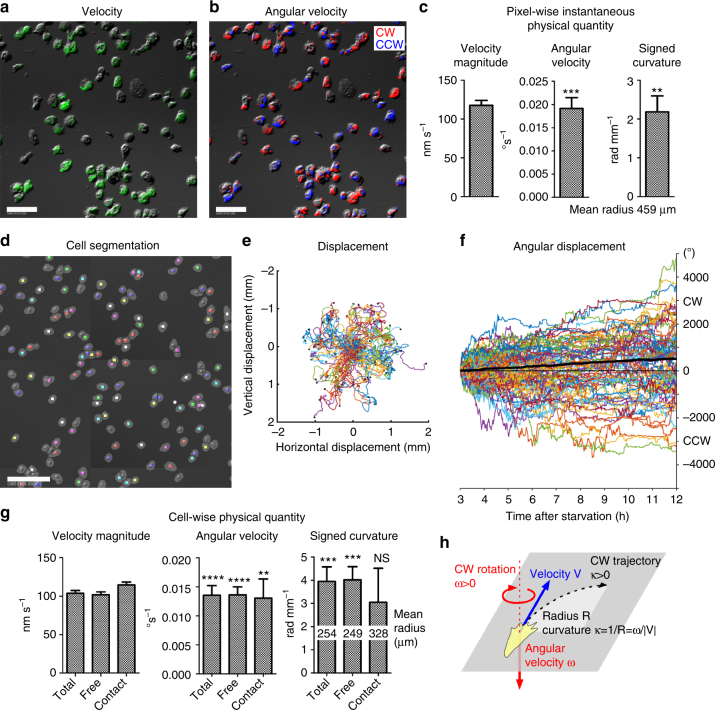
Motility analysis of *Dictyostelium* cell migration on 2D surface. **a**–**c** Pixel-wise analysis of single-field (330 × 330 μm) images from short-term (3–4 h after starvation; HAS) imaging of high-density (1 × 10^5^ cells per cm^2^) cultures. **a** Raw DIC image (gray) of cells at 3 HAS is overlaid by pixel-wise instantaneous velocity magnitude (green). **b** Distribution of angular velocity. Positive (red) and negative (blue) values represent clockwise (CW) and counterclockwise (CCW) rotation, respectively. Data from high-density culture #1 (**a**, **b**). **c** Summary of pixel-wise instantaneous physical quantities. The values represent mean ± s.e.m. of the means for individual cultures (*n* = 6). **P < 0.01, ***P < 0.001. One sample *t*-test for the null hypothesis that the mean is equal to zero. **d**–**g** Cell tracking analysis of multi-field (3.1 × 3.1 mm) stitched images from long-term (3–12 HAS) imaging of low-density (1 × 10^4^ cells per cm^2^) cultures. **d** Example of segmentation and tracking of cells at 7.68 HAS. Raw DIC image (gray) and smoothed RT-DIC images that were segmented into cell particles by watershed transform (gray ovals) are overlaid by optical flow-tracked cell centroids (seven different colors). **e**, **f** displacements **e** and angular displacements **f** for all tracked cells (*n* = 625) during 3–12 HAS. The mean angular displacement (black bold line) shows constant clockwise bias. Data from low-density culture #1 **d**–**f**. **g** Summary of cell-wise physical quantity. The quantities are collected in total frames or in the presence or absence of contacts with other cells. The values represent mean ± s.e.m. of the means for individual cultures (*n* = 8). **P < 0.01, ***P < 0.001, ****P < 0.0001, NS, *P* > 0.05. One sample *t*-test for the null hypothesis that the mean is equal to zero. **h** Rightward-biased clockwise migration on 2D surface revealed by the analysis. Scale bars, 50 μm in **a** and **b**, 200 μm in **d**

We further applied RT-DIC microscopy and the associated computational image analysis to assess the chirality in other cell types. We chose the cellular slime mold *Dictyostelium discoideum*, which is a unicellular organism that has been used as a model system to study cell migration and morphogenesis. Here we focused on their ameboid motion at the single-cell stage soon after starvation of nutrients. First, we performed single-shot and short-term (1 h) time-lapse imaging of 2D cultures at the density of 1 × 10^5^ cells per cm^2^ that is high but not eliciting cell aggregation. Raw DIC images visualized vigorous migration of the cells on 2D glass surface (Supplementary Movie 8). By visual inspection, we noticed that individual cells have the tendency of right-handed clockwise migration. To quantify the chiral pattern, DIC images were converted to RT-DIC intensity images. Using the RT-DIC images, pixel-wise instantaneous velocity (Fig. 6a, Supplementary Movie 8), angular velocity (Fig. 6b, Supplementary Movie 8) and signed curvature were calculated (Supplementary Fig. 7a–c, Supplementary Table 4). Summarized statistical data of the means for individual cultures show a significant clockwise bias in the angular velocity and the signed curvature (Fig. 6c).

Next, we analyzed the cell-wise motility by tracking of the cells from multi-field (3.1 × 3.1 mm areas) and long-term (9 h) DIC imaging of low-density (1 × 10^4^ cells per cm^2^) cultures. Cell particles were segmented by watershed transform of spatiotemporally smoothed RT-DIC images, and were tracked along successive frames by linking of their centroids with the aid of pixel-wise optical flow vectors (Fig. 6d, Supplementary Movie 9). The cell positions were tracked throughout the frames for 9 h (Fig. 6e). The cell trajectories visualized the tendency of clockwise migration at the level of individual cells (Supplementary Movie 10). The clockwise rotation of the cells was independent of each other. Spiral waves with concentric motion around singular points were not observed. The measurement of cumulative angular displacements quantitatively shows the constant clockwise bias (Fig. 6f). Physical quantities of the cells in this culture (Supplementary Fig. 7d–f) and all cultures (Supplementary Tables 5–7) indicate clockwise-biased angular velocity and curvature. We also evaluated the effect of cell–cell contacts on the clockwise migration by separately calculating the quantities in the presence or absence of contacts with other cells. The variance of the quantities was increased by cell–cell contacts, resulting in non-significant bias detection (Supplementary Tables 5–7). Summarized statistical data of the means for individual cultures indicate that clockwise bias was significant for angular velocity and signed curvature in all states, except for curvature in cell–cell contact state (Fig. 6g).

Taken together, RT-DIC imaging and the computational analysis revealed that *Dictyostelium* cells show the tendency of right-handed clockwise migration on 2D surface (Fig. 6h). They rotate clockwise at about 0.8° per min, producing one revolution per 7 h on average. The average curvature of the trajectory was about 4 rad per mm, forming a circle with about 250 μm radius.

### Slime mold cell protrusions rotate in right-screw direction

**Fig. 7:**
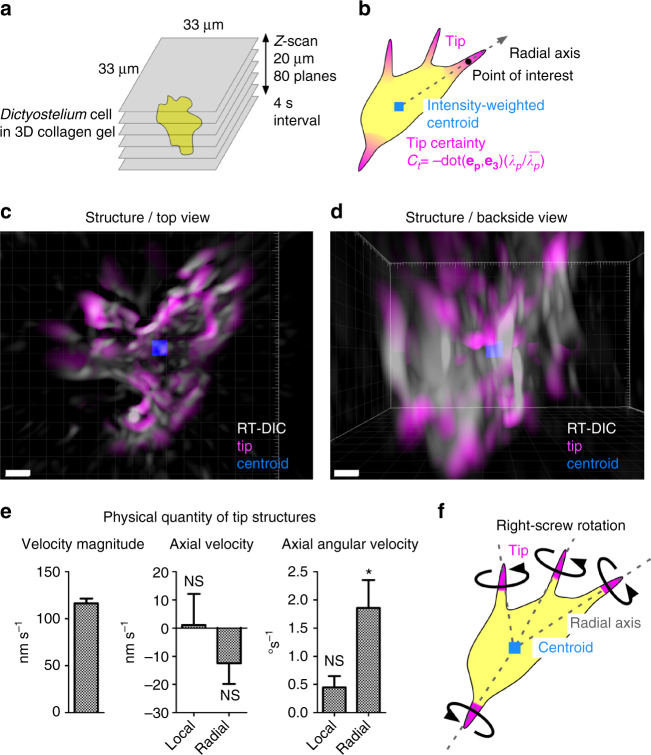
Whole-cell morphology and motility analyses of *Dictyostelium*. **a** Set-up for 3D time-lapse imaging. **b** Identification of tip structures from the tip certainty and calculation of tip motility along the radial axis from the intensity-weighted centroid. **c**, **d** Top **c** and backside **d** views of RT-DIC intensity (gray), intensity-weighted centroid (blue) and protrusions extracted by the tip certainty (magenta). Data from cell #1 **c**, **d**. **e** Summary of voxel-wise instantaneous tip motility. The values represent mean ± s.e.m. of the means for individual cells (*n* = 6). *P < 0.05, NS, *P* > 0.05. One sample *t*-test for the null hypothesis that the mean is equal to zero. **f** Right-screw rotation of tips about the radial axis revealed by the analysis. Scale bars, 5 μm

The chiral motility detected in *Dictyostelium* cells on 2D surface further raises the possibility that they also show chiral patterns at the subcellular levels. We performed 3D RT-DIC imaging and kinematic analysis in 3D collagen gel culture (Fig. 7a), and succeeded in capturing of whole images with RT-DIC imaging. The structure analysis detected the center of the cell as the intensity-weighted centroid and the protrusions as the tip certainty of the structure tensor (Fig. 7b, Supplementary Movie 11). In this study, we focused on fine protrusions including filopodia, but not on pseudopodia^42^, by setting the outer scale of the tensor, which is the diameter of interest, to about 0.6 μm. Time-lapse movies revealed a complex ameboid motion (Fig. 7c, d, Supplementary Movie 12). We quantified the motility of the protrusions by calculating the local instantaneous vector quantities and their scalar values projected onto the radial axis of the cell (Supplementary Fig. 8, Supplementary Tables 8, 9). Distribution pattern of radial angular velocity on the tip structure revealed the biased right-screw rotation along the radial axis (Supplementary Fig. 8e, Supplementary Table 9). Summarized statistical data show the significant right-handed rotation (Fig. 7e). These results indicate that individual protrusions of *Dictyostelium* cells tend to rotate in the right-screw direction about the cellular radial axis (Fig. 7f).

## Discussion

**Fig. 8:**
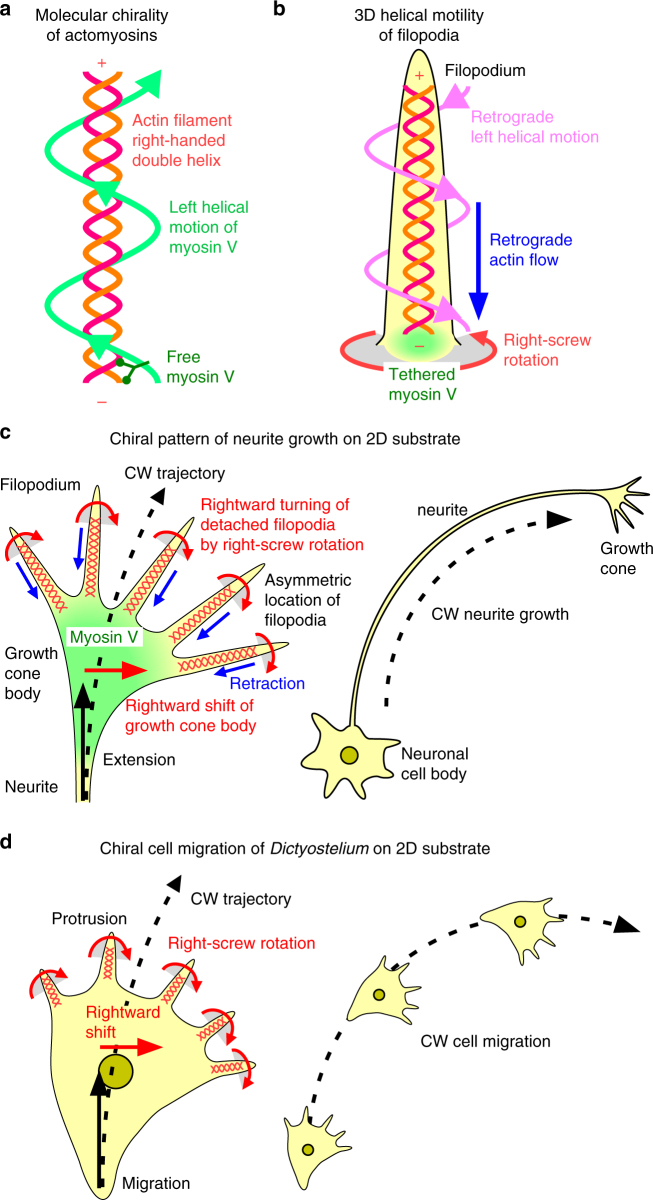
Schematic model of conversion from molecular chirality to chiral cell motility. **a** Molecular chirality is found in right-handed double helical structures of actin filaments and plus end-directed left-spiral motion of myosin V along actin filaments^49^. **b** In neuronal growth cone filopodia, actin filaments are enriched and polarized^51^. Myosin V is located at the base of filopodia^50^. We have revealed 3D left-helical motion of filopodia. The tethered myosin V would generate the left-helical motion by pulling back actin filaments and rotating them in the right-screw direction. **c** On 2D substrates, filopodia that are detached from substrates would rotate and attach to the substrates again on the right side. Rightward-biased asymmetric location of filopodia causes rightward traction of growth cone body^6^. As a result, neurites show clockwise growth^5,6^, which was quantified in the present study. **d** We have revealed that *Dictyostelium* cell protrusions including actin-rich filopodia rotate in the right-screw direction, though responsible myosin motor is yet unknown. As in the case of growth cone, on 2D substrates, the right-screw rotation of protrusions would generate rightward shift of the cell body. This shift would cause clockwise cell migration, which was demonstrated in this study

In the present study, we first developed two essential techniques for investigation of 3D cell structure, motion and their chirality. One is RT-DIC microscopy for precise 3D visualization with low phototoxicity, and the other is computational analysis for complex morphology and motility. With these techniques, we unveiled chiral motility in distinct cell types: 3D left-helical motion of neuronal growth cone filopodia with retraction and right-screw rotation; 2D right-handed clockwise migration and 3D right-screw rotation of protrusions in the cellular slime mold (Fig. 8).

RT-DIC microscopy retains the excellent properties of DIC microscopy that are suitable for live cell imaging, such as generation of high contrast in unstained specimens, optical sectioning with high-axial resolution, absence of halos, and low phototoxicity even with continuous illumination^21^. Importantly, this method does not generate problematic shadow-cast images, but produces unshaded self-luminous intensity images that can be treated with voxel- and intensity-based image processing and easily visualized with 3D reconstruction. RT-DIC microscopy allows 3D live imaging and quantitative analysis of fragile, photosensitive structures that were previously inaccessible with fluorescence imaging. Although RT-DIC microscopy with single-shot imaging is not suitable for complete restoration of shear-independent rotation-invariant images like the techniques with multi-shot imaging^23–25^, it is highly advantageous for rapid capture of 3D live images. In addition, RT-DIC microscopy uses a standard conventional DIC microscope and does not require any additional special equipment. RT can be applied to any DIC images with a simple code or plugin with one-step frequency filtering. The post-imaging transform enables offline analysis or reanalysis of previously accumulated DIC images. Thus, RT-DIC microscopy, as an alternative to fluorescence microscopy, will be a new standard technique for 3D visualization with a wide range of applications in the field of biological imaging.

To date, 3D cell motility has been investigated mostly by object-based image analyses^43–46^. They first delineate and extract cells as objects by image segmentation, and then quantify the shape and motion of the segmented cellular objects. Such object-based approach is certainly effective for the analysis of cells with simple morphology or spherical cell nuclei that can be represented with a few morphological parameters. In contrast, the analysis of cells with complex morphology is still challenging, though it has recently been achieved by application of specialized geometric techniques^47^. In this study, we adopted a different strategy that utilizes a voxel-based approach. At first, all of the basic quantities on the local geometric features and the physical motion are calculated for every voxel at every time. These quantities are not linked to any objects, but represented as sets of dense scalar, vector or tensor fields that are functions of space and time. Then, these local and instantaneous quantities are integrated along 3D space and time, and used for extraction of information on the 3D morphology and motility. Such approach enables to analyze complex morphology and ameboid motility of subcellular processes, as demonstrated in the neuronal growth cone and the cellular slime mold. Thus, the voxel-based techniques reported in this study will be widely applied to 3D motility analysis for various types of cells with diverse morphologies. In particular, these techniques will be effective tools for the analysis of rotational motility and its chiral or asymmetric nature in highly motile protrusions of the cells, such as the cilia, flagella, and filopodia.

The present techniques have revealed precise nature of 3D neuronal growth cone motility that could not be detected by conventional 2D imaging and manual analysis^6^. We have found that the trunk of filopodia exhibits a backward treadmill motion without net extension in 3D environments, which reflects the retrograde flow of actin filaments observed in 2D cultures^38^. We have further found that filopodia simultaneously show both overall retractions toward the growth cone body and right-screw rotation about their longitudinal axis, uncovering a left-helical chiral motion in 3D space (Fig. 8). This newly identified 3D left-helical motility is consistent with the findings that myosin V^48^, which drives the filopodial rotation^6^, is a left-spiral plus end-directed motor on the right-helical actin filaments^49^. Considering the facts that myosin V^50^ and the minus ends of actin filaments^51^ are located at the base of filopodia, it is plausible that the tethered myosin V simultaneously pulls and spins the polarized actin filaments. The established techniques and the detail information brought in this study will be the basis to resolve the molecular mechanisms that convert the chirality of actin filaments and myosin motors into the growth cone chirality and further into the chiral neuronal growth^5^. They will further provide a clue to elucidate their roles in the formation of left–right asymmetry in neural circuits and functional brain lateralization^1,2^, whose mechanism remains a complete puzzle.

We have demonstrated that the cellular slime mold *Dictyostelium discoideum* show two kinds of right-biased chiral motility at single-cell stages (Fig. 8). It is known that *Dictyostelium* cells form either clockwise or counterclockwise spiral pattern at the stages of aggregation and morphogenesis^52,53^, though an asymmetric bias was not reported. An intriguing question would be whether the single-cell chiral patterns affect the multicellular morphogenesis. The most important information brought by the findings on the slime mold is that the two phylogenetically distant species exhibit topologically similar chiral patterns with physical quantities in the same order. Both mammalian neuronal growth cones and the cellular slime mold show right-handed clockwise migration on 2D surface, forming a circle with hundreds of μm radius. Both further show right-screw rotation of their protrusive components in 3D environments with an angular speed of about a few degrees per second. Conserved principles, though not necessarily implemented with the same molecular machinery, may exist among different species for the conversion of the molecular chirality into the cellular chirality at the initial step of left–right asymmetry formation.

## Methods

### Neuronal cultures

For 3D dissociation cultures, hippocampal neurons were isolated from ICR mice at embryonic day 15, embedded in collagen gels at a density of 1–4 × 10^7^ cells per ml, and cultured at 37 °C overnight in glass-bottom dishes in phenol red-free L15 medium (Invitrogen) supplemented with penicillin and streptomycin, B27 (Invitrogen), and 10% FBS^6^. For 2D cultures, the dissociated neurons were re-aggregated overnight in 96-well plates (Lipidure-coat plate A-U96, NOF), plated onto coverslips coated with poly-d-lysine and laminin, and cultured for 48 h in L15 supplemented with penicillin, streptomycin and B27^6^. All the animal experiments were conducted in compliance with the protocol which was reviewed by the Institutional Animal Care and Use Committee and approved by the President of Niigata University (Permit Number: 82–2).

### *Dictyostelium* cell cultures

Axenic AX2 strain of the cellular slime mold *Dictyostelium discoideum* was provided by National BioResource Project. The cells were cultured for growth in HL5 medium at 22 °C^54^. For 2D culture, the cells in growth phase were washed with phosphate buffer (PB) and then plated onto glass-bottom dishes with PB at a density of 1 × 10^5^ cells per cm^2^ for pixel-wise analysis or 1 × 10^4^ cells per cm^2^ for cell tracking analysis. For 3D culture, the washed cells were embedded in collagen gels at a density of 1 × 10^6^ cells per ml and cultured in glass-bottom dishes with PB for 1–2 days.

### DIC imaging

A conventional standard upright microscope (BX61WI, Olympus) equipped with a water immersion objective and DIC optical components was used for observation^21^. The shear axis of the DIC prism (U-DICT) was oriented at a 45° angle. For live imaging, the microscope was equipped with an electric motor-driven XY stage (MD-XY30100T, Sigma Koki), a piezo motor-driven Z stage (99A604, Ludl) and a heat chamber (INU-UKOR-OS, Tokai Hit) in which the glass-bottom dish with the cultured cells was placed. In 3D neuronal cultures, growth cones extending toward the objective were chosen for imaging. DIC images (64.5 nm pitch) were acquired through a ×100 lens (NA 1.0) with a 12-bit CCD camera (ORCA-R2, Hamamatsu Photonics) under the control of MetaMorph software (Universal Imaging). Image planes at an interval of 250 nm in depth were scanned every second while changing the piezo focus under continuous illumination. The dimensions of the regions of interest were 16.5 μm (X) × 16.5 μm (Y) × 10 μm (Z). 3D *Dictyostelium* cell images were similarly acquired, but with 4 s intervals and dimensions of 33 μm (X) × 33 μm (Y) × 20 μm (Z). For 2D neurite cultures, still images were acquired through a ×20 lens (NA 0.5) from multiple visual fields of paraformaldehyde-fixed samples. For pixel-wise analysis of 2D *Dictyostelium* cultures, images were acquired through the ×20 lens from a single-visual field (330 × 330 μm, 322.5 nm pitch) with 10 s intervals during 3–4 HAS. For cell tracking analysis of 2D *Dictyostelium* cultures, images were acquired through a ×10 lens (NA 0.3) from multiple visual fields (3.1 × 3.1 mm area after stitching of 4 × 6 fields, 2.58 μm pitch after 4 × binning) with 30 s intervals during 3–12 HAS. The sequence of image stacks was saved as serially numbered multi-page TIFF files.

### Image processing

DIC image data were pre-processed, Riesz transformed, and post-processed with MATLAB (Mathworks) (Supplementary Fig. 4, Supplementary Software 2). In the pre-processing, the intensity was expanded to a full 12-bit range. Translational shifts between two successive image planes were detected by symmetric phase-only matched filtering^55^. The mean values along the *Z*-axis were defined as the *XY* stage drifts and used for their cancellation. Positioning errors along the *Z*-axis were corrected so that the mean square difference between the current intensity and the interpolated intensity was minimized. The pre-processed image data were converted by HT or RT (Supplementary Note 1). The transform was implemented with the 2D fast Fourier transform, multiplication by coefficients in the frequency domain, and the 2D inverse fast Fourier transform. The optimal filter was composed of a 45°-directed first-order RT, a 135°-directed second-order RT, and a high-pass filter that cuts direct-current (DC) and low-frequency components for flat-field correction^21^. The transformed images were post-processed for noise reduction and voxel interpolation. All 3D images were deconvoluted with open-source software DeconvolutionLab2^56^ with theoretical point spread function (PSF) based on Born–Wolf model generated by PSF Generator^57^. Both 2D and 3D images were smoothed with a Gaussian filter along the *X*, *Y*, and time axes. 3D images were upsampled by fourfold and interpolated along the *Z*-axis for conversion from anisotropic to isotropic cubic voxels. The intensity was again expanded to a 12-bit range. The final 3D RT-DIC image datasets had a pitch of 64.5 nm (X) × 64.5 nm (Y) × 62.5 nm (Z). Time resolution was 1 s for growth cone and 4 s for *Dictyostelium*. The 4D data sets were visualized by production of 2D cross sectional images, 3D rendered images, and movies with Imaris software (Bitplane).

### Image analysis

The structure and the motion of objects in image data sets were analyzed using voxel-wise (pixel-wise for 2D data) and intensity-based techniques of computer vision (Supplementary Fig. 4). All voxel-wise image data from all frames were used for the analysis. The structure was estimated by the structure tensor^35^, which is the second moment matrix of intensity (Supplementary Note 2, Supplementary Software 3). The local structure tensor was calculated for each voxel. The outer scale of the tensor was set to the size of interest. The direction vector that points to the distal tip of filopodia was calculated from the tensor-derived local fiber orientation. The certainty indices of fiber and distal tip structures were also calculated from the tensor. Each voxel was tracked backward with the direction vector. The retrograde tractability with the fiber direction was used for extraction of filopodia.

Voxel-wise displacement vectors between two image frames were estimated by optical flow analysis^36,37^ (Supplementary Note 3, Supplementary Software 4). The original Horn–Schunck method for dense 2D optical flow^36^ was modified for 3D images and for coarse-to-fine multi-scale estimation with Gaussian image pyramids^58^. For each voxel and each time point, five sequential image frames were used for estimation of four sets of displacement vectors (single-frame forward, single-frame backward, double-frame forward, and double-frame backward vectors). Velocity, acceleration, jerk, and angular velocity vectors were calculated from these displacement vectors. Axial components of velocity and angular velocity were calculated by their projections onto the structure tensor-derived direction vectors. Thresholding with the intensity, the structure indices, the fiber tractability and the velocity magnitude was used for segmentation of the voxels that were used for data presentation and the statistical analyses.

For cell tracking analysis of 2D *Dictyostelium* cultures, RT-DIC intensity images were spatiotemporally smoothed and then segmented into cell particles by watershed transform. The centroids of the cell particles in successive frames were tracked by linking with the aid of optical flow displacement vectors.

### Statistical analysis

The individual data were collected from large samples of each experimental trial, and were represented as mean ± s.d. The individual data were tested for difference from zero by the non-parametric Wilcoxon signed-rank test with the null hypothesis that the value of the median is equal to zero. All values and test results were shown in Supplementary Tables 1–9 and Fig. 5f. The summary data were the means of the large-sample individual data among different experimental trials, and were shown as mean ± s.e.m. They were tested with the parametric one sample *t*-test against zero value with the null hypothesis that the mean value is equal to zero. All values and test results were shown in Figs. 4f, 6c, 6g, 7e.

### Data availability

Some data sets are provided as Supplementary Data 1. The other data sets are available from the authors upon request. An ImageJ plugin for RT-DIC conversion is provided as Supplementary Software 1. MATLAB codes are provided as Supplementary Software 2–4.

## Electronic supplementary material

Supplementary Information

Peer Review File

Descriptions of Additional Supplementary Files

Supplementary Data 1

Supplementary Movie 1

Supplementary Movie 2

Supplementary Movie 3

Supplementary Movie 4

Supplementary Movie 5

Supplementary Movie 6

Supplementary Movie 7

Supplementary Movie 8

Supplementary Movie 9

Supplementary Movie 10

Supplementary Movie 11

Supplementary Movie 12

Supplementary Software 1

Supplementary Software 2

Supplementary Software 3

Supplementary Software 4
