# Supplementary material for: Revealing chiral cell motility by 3D Riesz transform-differential interference contrast microscopy and computational kinematic analysis
Source: Nat Commun. 2017 Dec 19;8:2194. doi: 10.1038/s41467-017-02193-w (PMC5736583; doi:10.1038/s41467-017-02193-w)
Supplement: Supplementary file 1 — Supplementary Information [file 41467_2017_2193_MOESM1_ESM.pdf]

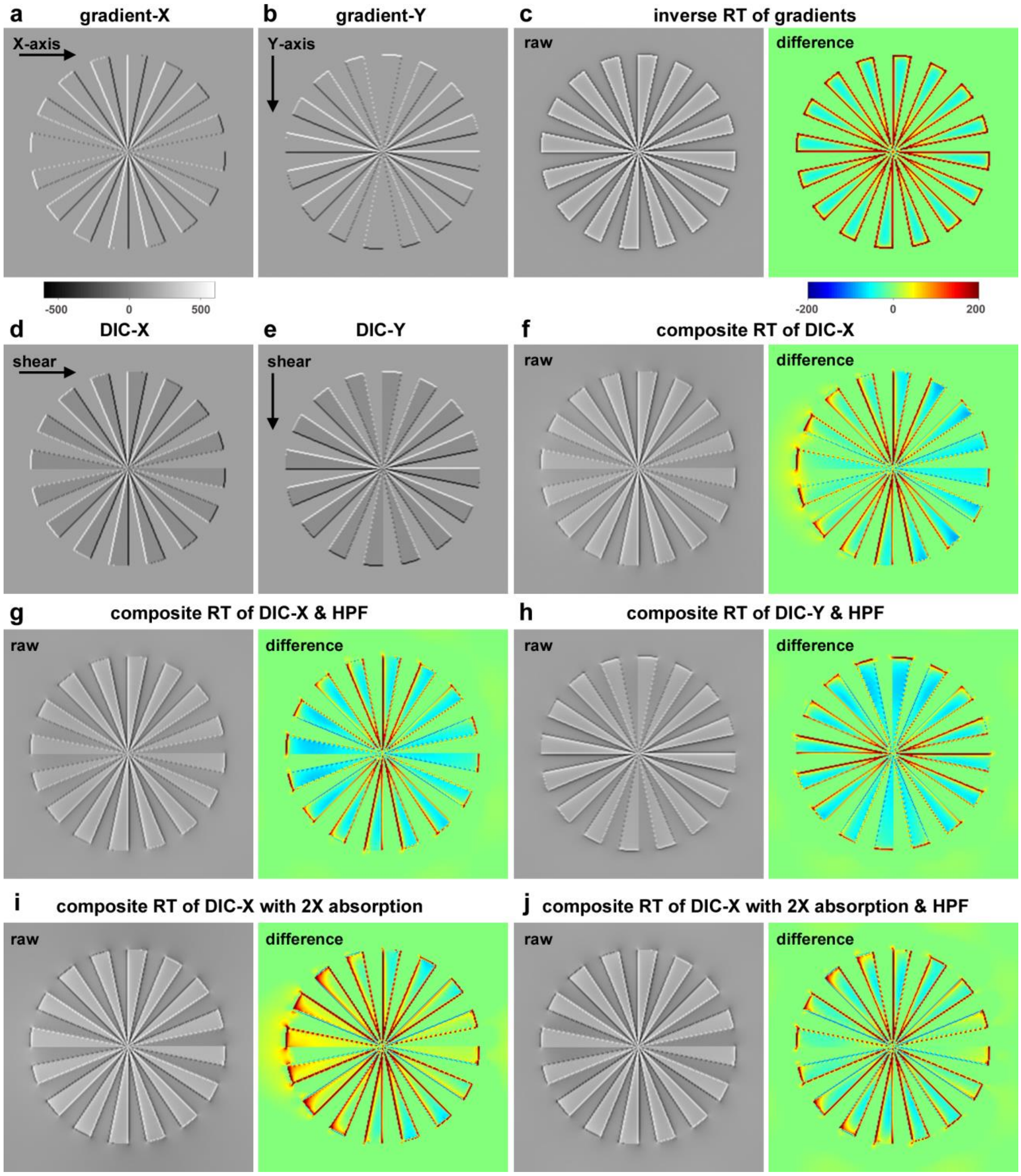

Supplementary Figure 1 Comparison of the single-shot composite RT with the multi-shot inverse RT[1, 2, 3] for DIC image restoration. (**a,b**) Gradients of the ground truth (GT) image (256 × 256 pixels, **Fig. 1a**) along X-axis (**a**) and Y-axis (**b**). (**c**) Inverse RT of a complex image with **a** in the real and **b** in the imaginary components. Raw image (left) and difference between the positively thresholded image and the GT (right). Given the pure orthogonal gradients, the inverse RT restores an isotropic rotation-invariant edge-enhanced phase image. (**d,e**) DIC images synthesized by adding an inverted absorption image of the GT to the gradients (**a,b**). (**f**) Composite RT restored the GT directly from **d** alone, but it generated an anisotropic image with less sensitivity along the shear and diffuse artifacts around the object. (**g,h**) Composite RT and high-pass filtering (HPF, $\lambda < 256/3$ pixels) of **d** (**g**) or **e** (**h**) removed the diffuse artifacts. The anisotropy still remained. (**i,j**) Two-fold enhancement of the second-order RT compensated the lower sensitivity to the shear axis at the expense of an increase in the diffuse artifacts (**i**), which were largely suppressed by HPF (**j**).

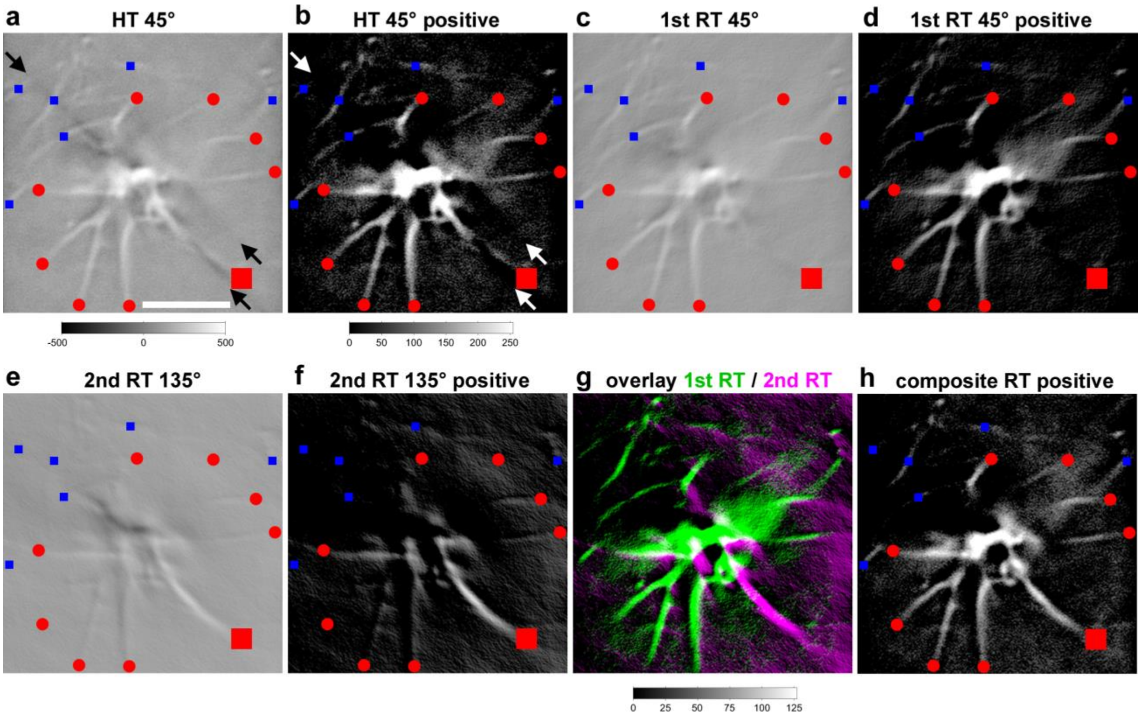

Supplementary Figure 2 Application of various Fourier transform-based phase conversion techniques to a DIC microscopic image of a growth cone. (**a-f**) 45°-directed HT (**a, b**), 45°-directed first-order RT (**c, d**) and 135°-directed second-order RT (**e, f**) of a DIC image (**Fig. 2b**). **b**, **d** and **f** are threholded images of **a**, **c** and **e** with positive values, respectively. Filopodia (red circles) and collagen fibers (blue squares) are visible. A filopodium directed to 45° (red square) seen in the original image (**Fig. 2b)** was artificially fragmented in **a** and **b**, invisible in **c** and **d**, but fully recovered in **e** and **f**. Note that line artifacts (arrows) were found in **a** and **b**, but not in the other transforms. (**g**) Overlay of **d** and **f**. (**h**) Thresholded image of composite RT (**Fig. 2e**). Note that shading bias in the upper-left quadrant of **Fig. 2e** was enhanced by the thresholding and appeared as dark area (**h**), and that it was corrected by high-pass filtering (**Fig. 2g**). Data from cell #2. Scale bar, 5 μm.

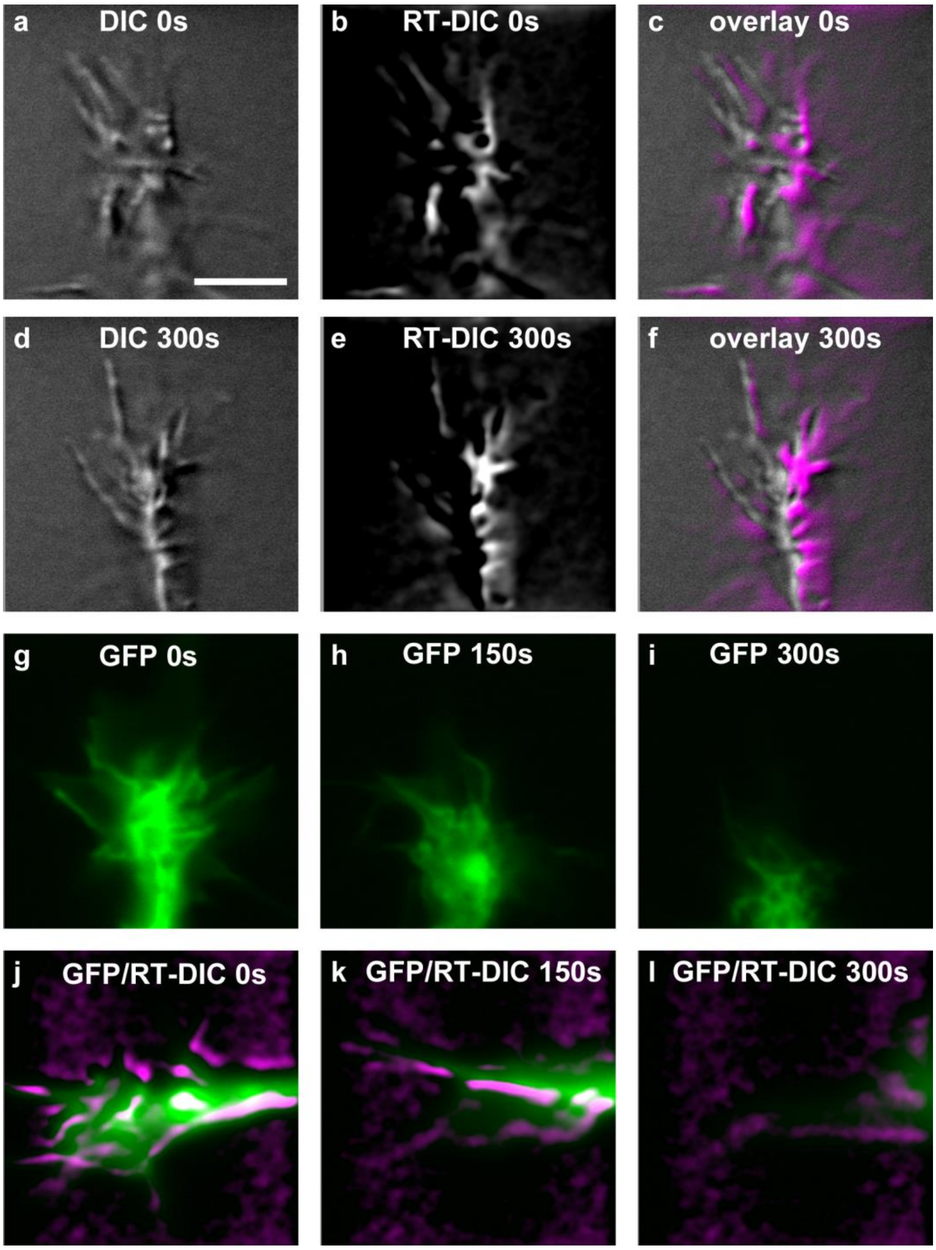

Supplementary Figure 3 Comparison between RT-DIC microscopy and fluorescence microscopy for frequent time-lapse imaging of neuronal growth cones. (**a-i**) Time-lapse images of a GFP-labeled growth cone sequentially acquired by 2D RT-DIC (**a-f**) and wide-field fluorescence (**g-i**) imaging (See **Supplementary Movie 1**). **a-c** and **d-f** are the first and last frames of RT-DIC imaging, respectively. The first (**g**), middle (**h**) and last (**i**) frames were shown for fluorescence imaging. The filopodial morphology and motility did not change during RT-DIC imaging, but were soon disorganized and collapsed after switching to fluorescence imaging even with low-power illumination. In addition, although RT-DIC intensity was constant, GFP intensity was gradually diminished. (**j-l**) Simultaneous RT-DIC and fluorescence imaging of another growth cone. As shown in the initial frame (**j**), GFP labeling reflects cytosolic volume, whereas RT-DIC tends to capture filopodia, which is rich in membrane. The growth cone retracted and collapsed during imaging. Scale bar, 5 μm.

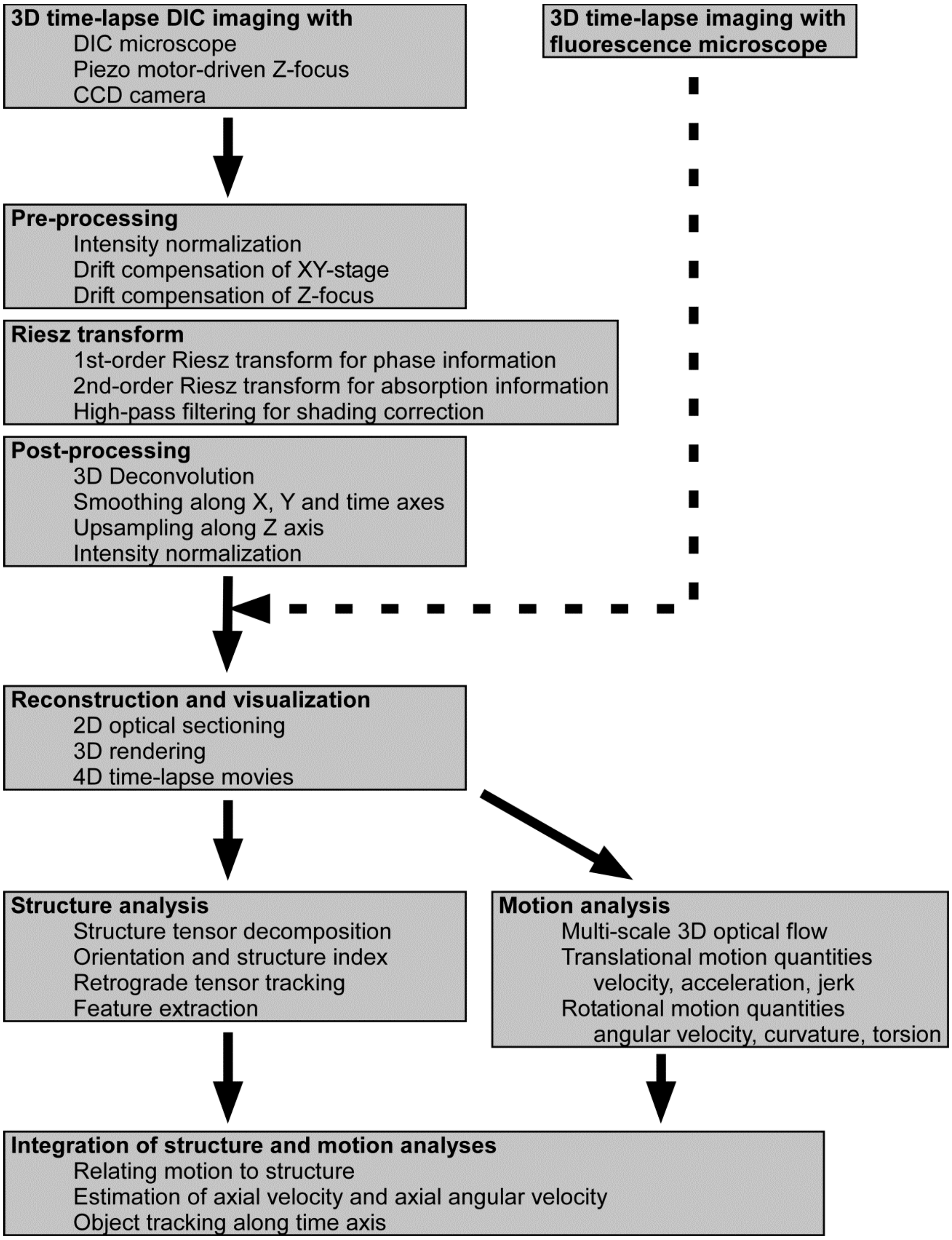

Supplementary Figure 4 A diagram of 3D RT-DIC time-lapse imaging and the succeeding image analyses of structure and motion.

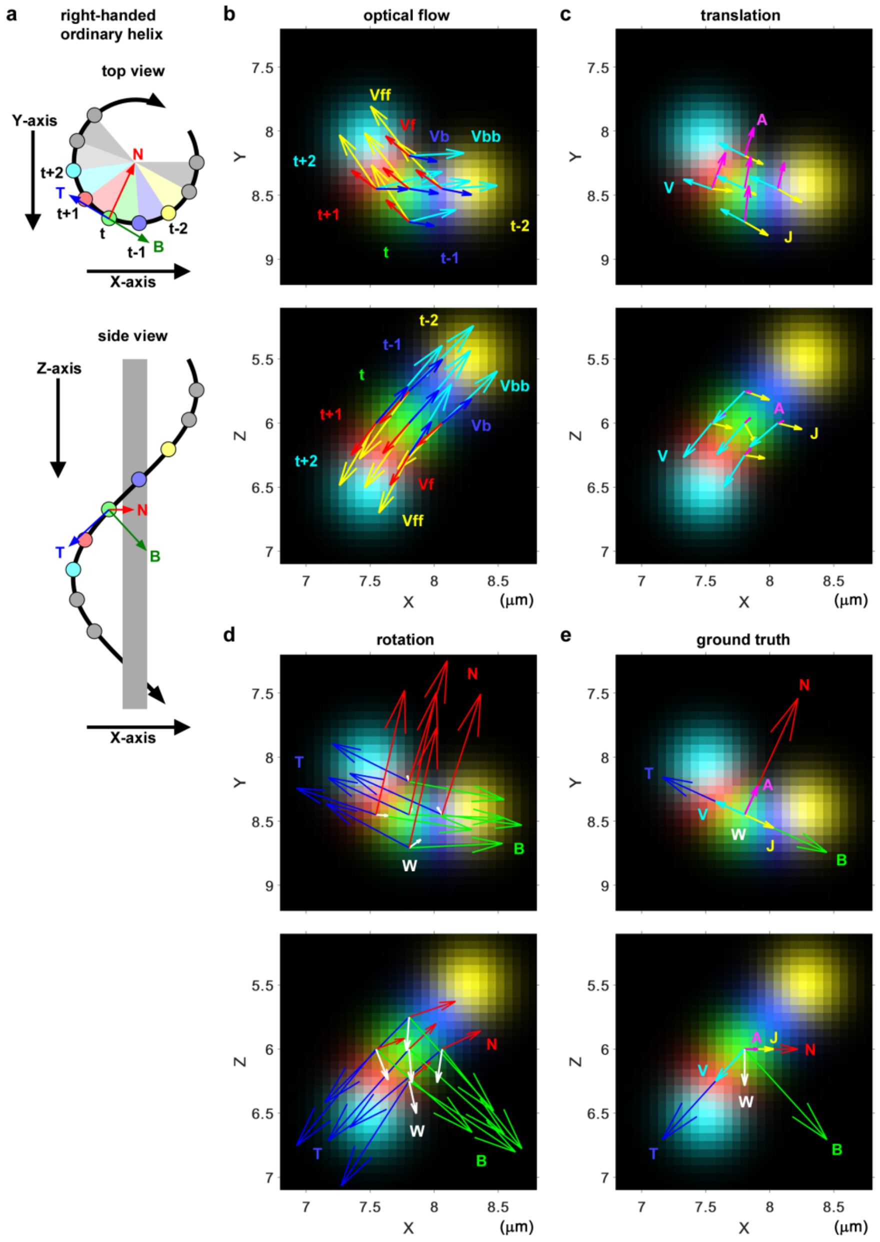

Supplementary Figure 5 Kinematic analysis of a synthetic sphere moving along a helical path. (**a**) A scheme showing the path of a sphere moving along a right-handed ordinary helix. TNB (tangent-normal-binormal) frames are shown on the sphere at time t. (**b-e**) XY plane (upper panels) and XZ plane (lower panels) projected images showing the trajectory of a Gaussian-blurred sphere along the helical path. The position of the sphere is given as $(x, y, z) = (a\cos wt, a\sin wt, bt)$, where $a = 500\ (\mathrm{nm\ s^{-1}})$, $\mathrm{b} = 500\ (\mathrm{nm\ s^{-1}})$ and $w = 0.5\ (\mathrm{rad\ s^{-1}})$. The sphere in each frame is shown with a different color. (**a**) Single-frame forward (Vf) and backward (Vb), and double-frame forward (Vff) and backward (Vbb) optical flow vectors from the central frame were plotted on the sphere image. (**b**) Estimated translational physical quantities (velocity, V; acceleration, A; and jerk, J). (**c**) Estimated rotational physical quantities (tangent, T; normal, N; binormal B; and angular velocity, W). (**d**) Ground-truth physical quantities. One unit in the axis represents 1×10^3 nm for position, 1×10^3 nm s^{-1} for velocity, 5×10^2 nm s^{-2} for acceleration, 2.5×10^2 nm s^{-3} for jerk, 1 for TNB frames, 2 rad s^{-1} (57.3 ° s^{-1}) for angular velocity.

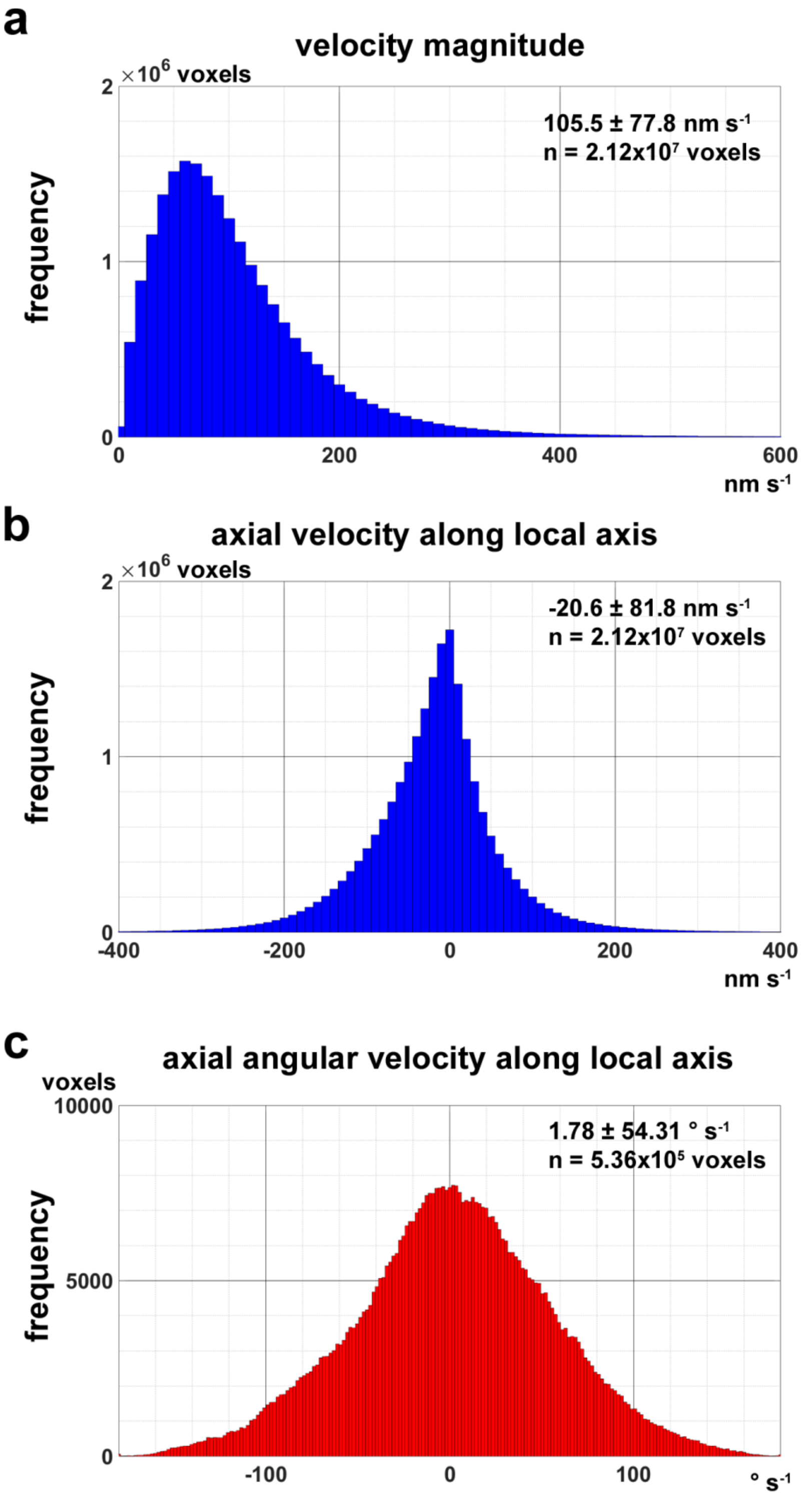

Supplementary Figure 6 Examples of histograms showing voxel-wise instantaneous physical quantities of the growth cone motility. Velocity magnitude (**a**), local axial velocity (**b**) and local axial angular velocity (**c**) for all voxels in all frames in the images of the growth cone from cell #2 (**Supplementary Table 2** and **3**). The values represent the mean ± s.d with replication numbers of voxels.

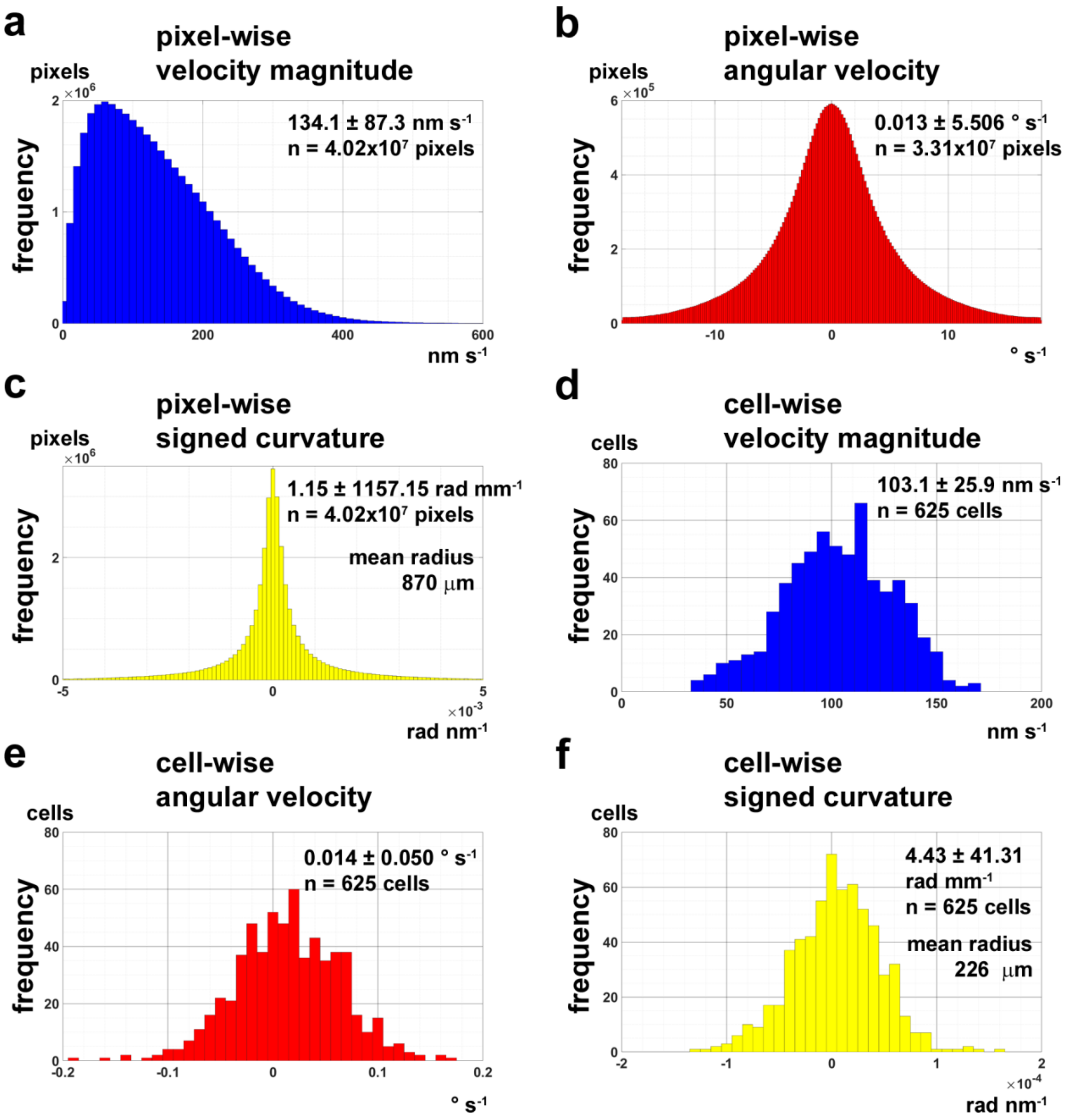

Supplementary Figure 7 Examples of histograms showing physical quantities of *Dictyostelium* cell migration on 2D glass surface. (**a-c**) Pixel-wise instantaneous velocity magnitude (**a**), angular velocity (**b**) and signed curvature (**c**) for all pixels in all frames in the images from culture #1 (**Supplementary Table 4**). (**d-f**) Cell-wise velocity magnitude (**d**), angular velocity (**e**) and signed curvature (**f**) for all tractable cells in culture #1 (**Supplementary Table 5-7**). The values represent the mean ± s.d. with replication numbers of pixels or cells.

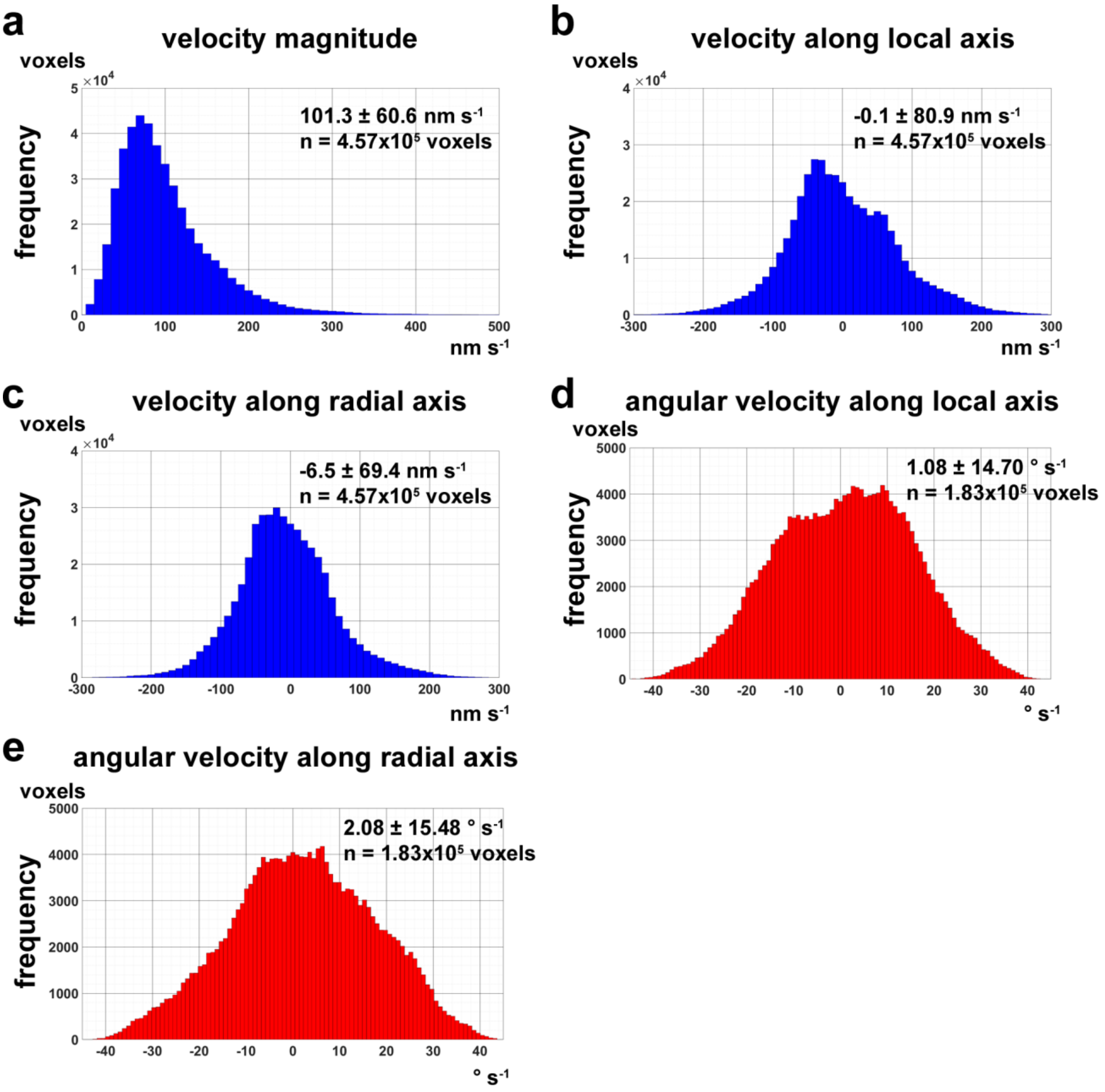

Supplementary Figure 8 Examples of histograms showing voxel-wise instantaneous physical quantities of *Dictyostelium* cell protrusions in 3D collagen gels. Velocity magnitude (**a**), local axial velocity (**b**) and local axial angular velocity (**c**) for all voxels in all frames in the images of cell #1 (**Supplementary Table 8** and **9**). The values represent the mean ± s.d with replication numbers of voxels..

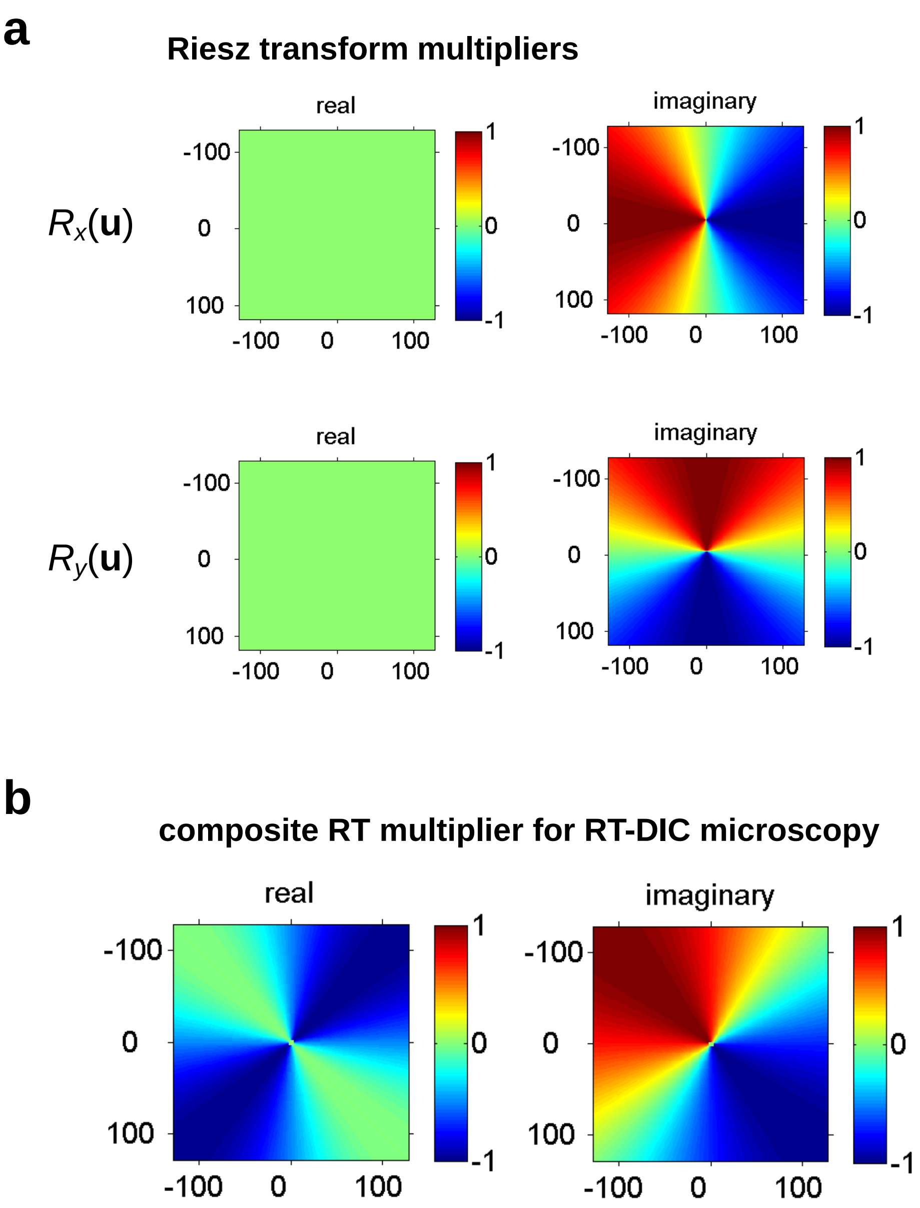

Supplementary Figure 9 RT multipliers in the frequency domain. (**a**) A set of discrete RT multipliers for 256 × 256 pixel images. (**b**) Composite multiplier for RT-DIC microscopy. An example with $N_{pixel} = 256, \theta_1 = 45°, \theta_2 = 135°, u_{cut} = 3/N_{pixel}$. See **Supplementary Note 1** for details.

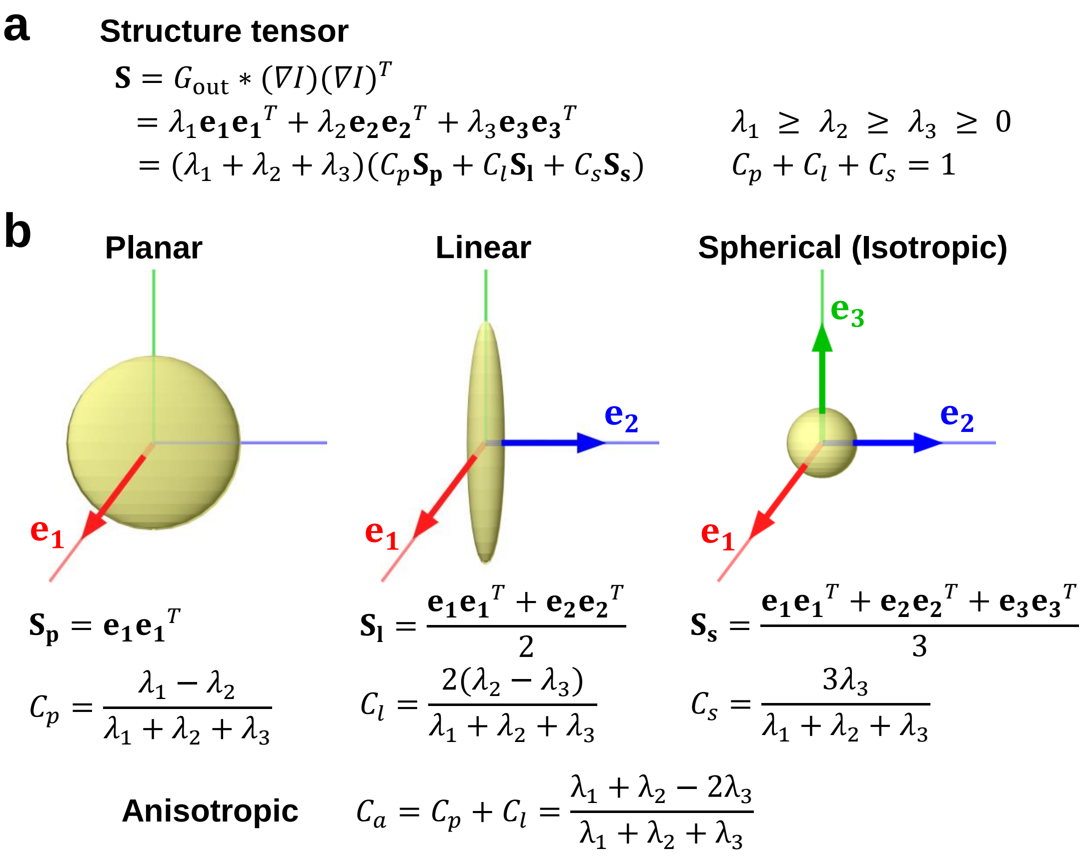

Supplementary Figure 10 Shape classification of the structure tensor. (**a**) Calculation and decomposition of the structure tensor. (**b**) Shape of the three tensor bases and the certainty indices. See **Supplementary Note 2** for details.

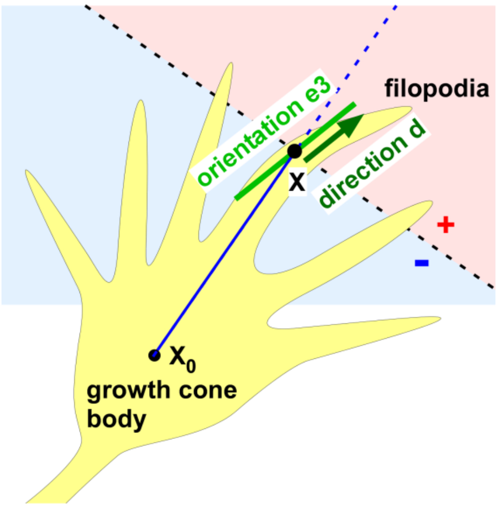

Supplementary Figure 11 Conversion of orientation to direction. The direction of the filopodium is determined by the local orientation and the relative position from the center of the growth cone body. See **Supplementary Note 2** for details.

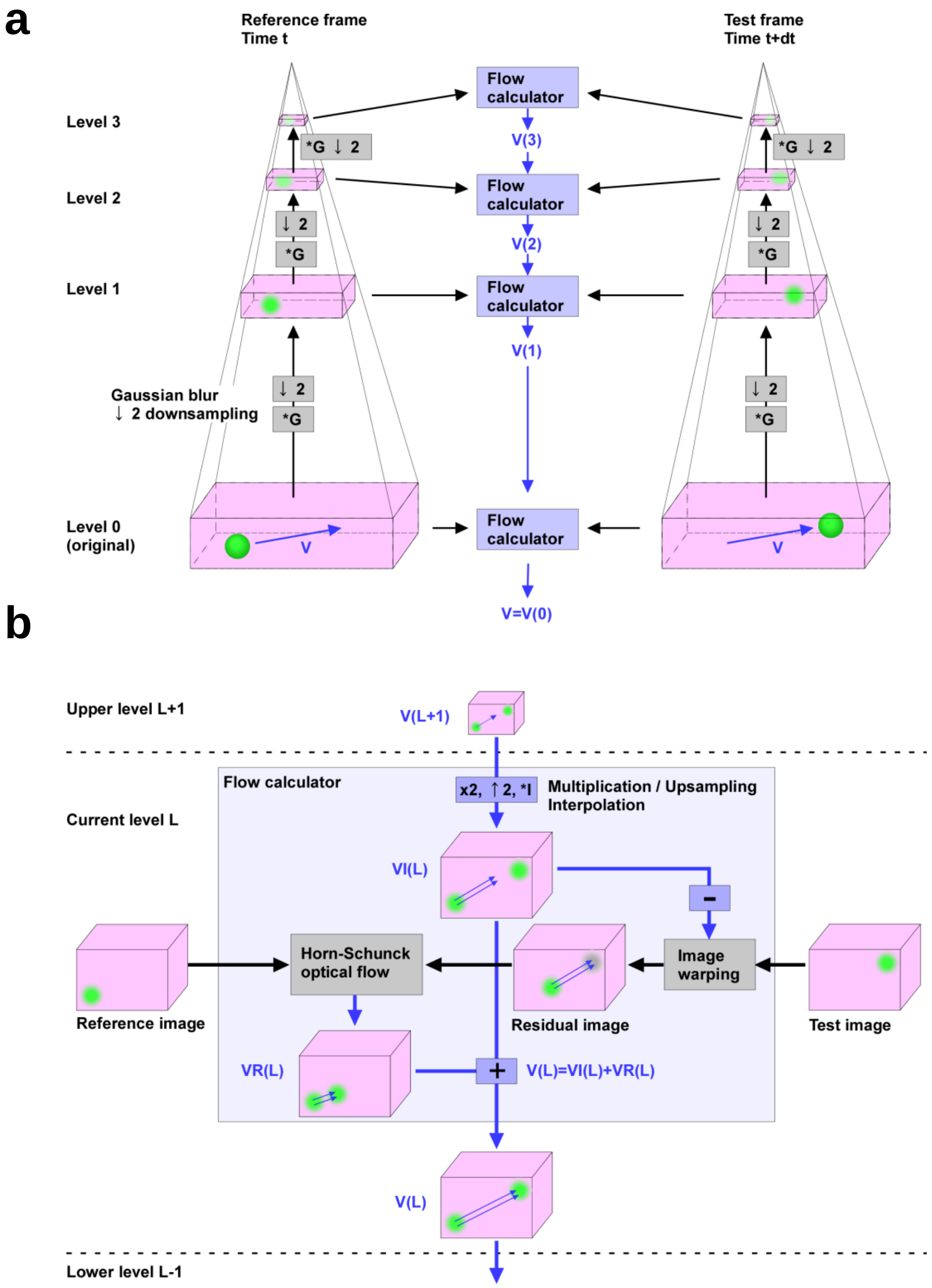

Supplementary Figure 12 Coarse-to-fine multi-scale optical flow. (**a**) A schematic for construction of Gaussian pyramids and calculation of optical flow. (**b**) Protocol for optical flow calculation at each level of the pyramid. See **Supplementary Note 3** for details.

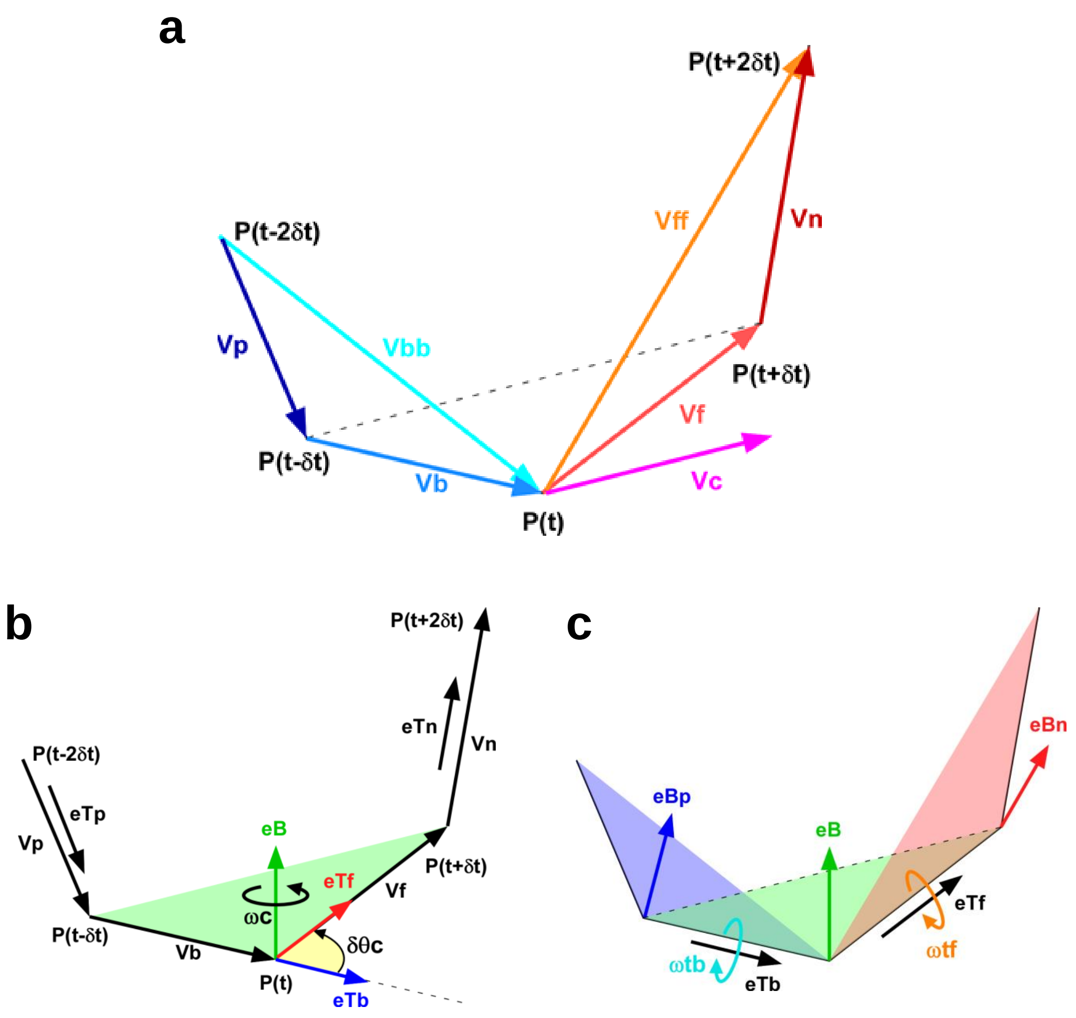

Supplementary Figure 13 Estimation of voxel-wise kinematics from optical flow vectors. (**a**) Estimation of translational velocity. Single- and double-frame optical flow analyses estimate four velocities; **Vf**, **Vb**, **Vff** and **Vbb**. They are used for derivation of next **Vn**, previous **Vp** and central **Vc** velocities. (**b,c**) Estimation of Frenet-Serret TNB frames and angular velocities. Tangent, binormal and normal vectors are derived from the four velocity vectors. Curvature angular velocity is calculated as the rotation speed of the tangent about the binormal vector (**b**). Torsional angular velocity is calculated as the rotation speed of the binormal vector about the tangent vector (**c**). See **Supplementary Note 3** for details.

Supplementary Table 1 Physical quantities of synthetic helical motion estimated by kinematic analysis.

**physical quantity**	**ground truth**	**magnitude** mean ± s.d. (%)	**endpoint error** (%)	**angular error** (°)
**velocity**	353.6 nm s^{-1}	92.8 ± 9.1	14.0	5.1
**acceleration**	125.0 nm s^{-2}	104.7 ± 14.3	29.8	14.6
**jerk**	62.5 nm s^{-3}	80.9 ± 12.5	40.7	21.0
**angular velocity**	28.6 ° s^{-1}	95.4 ± 5.5	28.8	16.4

The values were calculated for all the voxels in the sphere (n = 613 voxels).

Supplementary Table 2 Voxel-wise instantaneous velocities of growth cone filopodia estimated by kinematic analysis.

**cell**	**velocity** (nm s^{-1})				
	**voxel**	**magnitude**	**local axis**	**chordal axis**	**orbital axis**
1	$2.80{\times}10^{7}$	84.2 ± 72.8	-14.0 ± 71.4†	-11.7 ± 70.8†	-13.1 ± 72.7†
2	$2.12{\times}10^{7}$	105.5 ± 77.8	-20.6 ± 81.8†	-19.4 ± 83.1†	-19.0 ± 82.9†
3	$1.35{\times}10^{7}$	105.3 ± 85.2	-12.0 ± 81.1†	-8.4 ± 81.4†	-11.2 ± 84.2†
4	$1.12{\times}10^{7}$	71.8 ± 48.9	-16.3 ± 57.3†	-14.1 ± 56.6†	-16.5 ± 58.8†
5	$2.08{\times}10^{7}$	69.6 ± 50.6	1.1 ± 56.8†	0.4 ± 55.8†	1.2 ± 56.2†
6	$1.65{\times}10^{7}$	106.0 ± 86.5	-4.6 ± 83.8†	-5.1 ± 82.7†	-5.4 ± 84.7†
7	$1.62{\times}10^{7}$	113.3 ± 94.8	-15.0 ± 86.7†	-12.9 ± 87.5†	-12.9 ± 90.3†
8	$2.12{\times}10^{7}$	115.8 ± 82.4	-31.8 ± 89.8†	-28.8 ± 89.6†	-31.6 ± 90.5†

The values represent mean ± s.d. for replication numbers of voxels shown in the left columns. $^{\dagger}P<0.0001$. Wilcoxon signed rank test for the null hypothesis that the median is equal to zero. Cell #2 is presented in **Figs. 2, 3, 4** and **Supplementary Fig. 2**. Histograms of cell #2 data are shown in **Supplementary Fig. 6**.

Supplementary Table 3 Voxel-wise instantaneous angular velocities of growth cone filopodia estimated by kinematic analysis.

**cell**	**angular velocity** (° s^{-1})				
	**voxel**	**magnitude**	**local axis**	**chordal axis**	**orbital axis**
1	$5.20{\times}10^{5}$	76.6 ± 37.2	1.50 ± 52.01†	1.14 ± 51.67†	1.30 ± 53.29†
2	$5.36{\times}10^{5}$	78.4 ± 37.9	1.78 ± 54.31†	1.17 ± 54.45†	1.35 ± 54.95†
3	$4.42{\times}10^{5}$	77.8 ± 38.3	1.03 ± 51.86†	0.92 ± 51.05†	0.33 ± 52.66†
4	$4.0{\times}10^{4}$	71.0 ± 40.0	1.21 ± 46.78†	1.59 ± 48.58†	0.95 ± 51.96†
5	$9.6{\times}10^{4}$	83.1 ± 37.9	0.87 ± 52.01†	0.54 ± 52.27†	0.90 ± 52.67†
6	$5.88{\times}10^{5}$	81.3 ± 37.2	0.51 ± 52.14†	0.23 ± 52.10$^{n.s.}$	-0.05 ± 53.19†
7	$7.62{\times}10^{5}$	76.3 ± 38.8	-0.03 ± 49.21†	-0.39 ± 50.07**	-0.14 ± 51.62$^{n.s.}$
8	$6.21{\times}10^{5}$	78.3 ± 38.4	2.45 ± 55.57†	2.51 ± 55.11†	3.39 ± 55.85†

The values represent mean ± s.d. for replication numbers of voxels shown in the left columns. $^{**}P<0.01$, $^{\dagger}P<0.0001$, $^{n.s.}P>0.05$. Wilcoxon signed rank test for the null hypothesis that the median is equal to zero. Cell #2 is presented in **Figs. 2, 3, 4** and **Supplementary Fig. 2**. Histograms of cell #2 data are shown in **Supplementary Fig. 6**.

Supplementary Table 4 Pixel-wise instantaneous physical quantities of *Dictyostelium* cell migration on 2D glass surface.

culture	translation		rotation			
	**pixel**	**velocity magnitude** (nm s^{-1})	**pixel**	**angular velocity** (° s^{-1})	**signed curvature** (rad mm^{-1})	**radius** (μm)
1	4.02×10^7	134.1 ± 87.3	3.31×10^7	0.013 ± 5.506†	1.15 ± 1157.15†	870
2	4.34×10^7	110.2 ± 81.6	3.18×10^7	0.028 ± 5.978†	3.17 ± 1287.46†	315
3	3.95×10^7	128.5 ± 88.4	3.14×10^7	0.019 ± 5.534†	3.54 ± 1151.08†	282
4	4.22×10^7	106.5 ± 80.0	3.06×10^7	0.022 ± 5.631†	2.32 ± 1240.85†	431
5	4.43×10^7	129.9 ± 84.4	3.62×10^7	0.019 ± 5.383†	1.75 ± 1136.35†	571
6	5.18×10^7	97.4 ± 72.0	3.63×10^7	0.013 ± 5.798†	1.18 ± 1321.86†	847

The values represent mean ± s.d. for replication numbers of pixels shown in the left columns. †P<0.0001. Wilcoxon signed rank test for the null hypothesis that the median is equal to zero. Culture #1 is shown in **Fig. 6a, b**. Histograms of culture #1 data are shown in **Supplementary Fig. 7**.

Supplementary Table 5 Cell-wise velocities of *Dictyostelium* cell migration on 2D glass surface.

culture	tracked cells	velocity magnitude (nm s^{-1})			free / total duration (%)
		**total**	**free**	**contact**	
1	625	103.1 ± 25.9	101.2 ± 26.7	112.0 ± 26.4	79.2
2	663	102.9 ± 27.1	101.6 ± 28.3	108.9 ± 26.4	76.6
3	521	93.6 ± 24.6	92.5 ± 25.2	101.9 ± 27.4	83.4
4	399	83.7 ± 28.5	81.3 ± 29.8	102.3 ± 32.4	81.4
5	413	116.4 ± 30.8	114.2 ± 31.9	129.0 ± 28.7	82.0
6	521	107.7 ± 28.5	105.3 ± 29.7	118.8 ± 29.0	78.1
7	435	112.3 ± 30.4	110.0 ± 31.2	126.8 ± 32.8	81.7
8	430	109.0 ± 30.0	108.1 ± 30.7	117.1 ± 31.6	82.3

The values represent mean ± s.d. for replication numbers of cells shown in the left columns. Culture #1 is shown in **Fig. 6d-f**. Histograms of culture #1 data are shown in **Supplementary Fig. 7**.

Supplementary Table 6 Cell-wise angular velocities of *Dictyostelium* cell migration on 2D glass surface.

culture	tracked cells	angular velocity (° s^{-1})			free / total duration (%)
		**total**	**free**	**contact**	
1	625	0.014 ± 0.050†	0.015 ± 0.055†	0.013 ± 0.133*	78.9
2	663	0.014 ± 0.050†	0.014 ± 0.057†	0.016 ± 0.127**	76.2
3	521	0.013 ± 0.051†	0.012 ± 0.053†	0.014 ± 0.164 n.s.	83.0
4	399	0.003 ± 0.055 n.s.	0.005 ± 0.060 n.s.	-0.007 ± 0.167 n.s.	79.7
5	413	0.016 ± 0.048†	0.016 ± 0.053†	0.015 ± 0.154 n.s.	81.5
6	521	0.015 ± 0.057†	0.016 ± 0.062†	0.010 ± 0.143*	77.4
7	435	0.019 ± 0.053†	0.018 ± 0.056†	0.026 ± 0.159***	81.2
8	430	0.015 ± 0.046†	0.015 ± 0.050†	0.017 ± 0.156*	82.1

The values represent mean ± s.d. for replication numbers of cells shown in the left columns. †P<0.0001, ***P<0.001, **P<0.01, *P<0.05, n.s.P>0.05. Wilcoxon signed rank test for the null hypothesis that the median is equal to zero. Culture #1 is shown in **Fig. 6d-f**. Histograms of culture #1 data are shown in **Supplementary Fig. 7**.

Supplementary Table 7 Cell-wise signed curvatures of *Dictyostelium* cell migration on 2D glass surface.

culture	tracked cells	**signed curvature** (rad mm^{-1})			**free / total duration** (%)
		**total**	**free**	**contact**	
1	625	4.43 ± 41.31**	4.36 ± 47.12**	4.67 ± 88.19$^{n.s.}$	78.9
2	663	4.41 ± 40.64**	4.04 ± 46.55*	6.11 ± 88.37$^{n.s.}$	76.2
3	521	3.60 ± 41.05**	3.22 ± 43.69$^{n.s.}$	2.79 ± 105.15$^{n.s.}$	83.0
4	399	0.15 ± 47.70$^{n.s.}$	0.56 ± 51.56$^{n.s.}$	-3.66 ± 112.62$^{n.s.}$	79.7
5	413	4.67 ± 36.93*	5.09 ± 39.77*	3.60 ± 84.67$^{n.s.}$	81.5
6	521	4.59 ± 41.71**	5.68 ± 48.36*	0.54 ± 89.81$^{n.s.}$	77.4
7	435	6.34 ± 39.89**	5.06 ± 44.49*	10.02 ± 93.70**	81.2
8	430	3.36 ± 37.38$^{n.s.}$	4.12 ± 42.80$^{n.s.}$	0.34 ± 97.51$^{n.s.}$	82.1

The values represent mean ± s.d. for replication numbers of cells shown in the left columns. **P<0.01, *P<0.05, $^{n.s.}$P>0.05. Wilcoxon signed rank test for the null hypothesis that the median is equal to zero. Culture #1 is shown in **Fig. 6d-f**. Histograms of culture #1 data are shown in **Supplementary Fig. 7**.

Supplementary Table 8 Voxel-wise instantaneous velocities of *Dictyostelium* cell protrusion in 3D collagen gels.

cell	**tip velocity** (nm s^{-1})			
	**voxel**	**magnitude**	**local axis**	**radial axis**
1	4.57×10^{5}	101.3 ± 60.6	-0.1 ± 80.9†	-6.5 ± 69.4†
2	2.2×10^{4}	107.6 ± 77.6	-24.7 ± 80.9†	-25.9 ± 78.3†
3	2.52×10^{5}	128.1 ± 87.2	27.4 ± 107.0†	-12.5 ± 81.7†
4	1.04×10^{5}	110.3 ± 75.3	-18.0 ± 93.3†	-31.0 ± 72.6†
5	7.8×10^{4}	115.3 ± 73.1	-18.3 ± 95.3†	-18.4 ± 91.5†
6	4.7.4×10^{5}	134.3 ± 72.1	-40.4 ± 109.5†	19.5 ± 80.3†

The values represent mean ± s.d. for replication numbers of voxels shown in the left columns. †P<0.0001. Wilcoxon signed rank test for the null hypothesis that the median is equal to zero. Cell #1 is shown in **Fig. 7**. Histograms of culture #1 data are shown in **Supplementary Fig. 8**.

Supplementary Table 9 Voxel-wise instantaneous angular velocities of *Dictyostelium* cell protrusion in 3D collagen gels.

cell	**tip angular velocity** (° s^{-1})			
	**voxel**	**magnitude**	**local axis**	**radial axis**
1	1.83×10^{5}	24.7 ± 9.12	1.08 ± 14.70†	2.08 ± 15.48†
2	9.3×10^{3}	22.3 ± 10.36	0.10 ± 12.25†	4.06 ± 13.47†
3	1.27×10^{5}	21.1 ± 9.24	0.83 ± 13.29†	1.73 ± 13.85†
4	4.7×10^{4}	25.3 ± 8.89	-0.26 ± 15.36†	1.52 ± 15.35†
5	3.6×10^{5}	25.7 ± 9.90	0.62 ± 15.04†	1.29 ± 15.33†
6	2.99×10^{5}	24.7 ± 8.76	0.32 ± 16.02†	0.44 ± 15.52†

The values represent mean ± s.d. for replication numbers of voxels shown in the left columns. †P<0.0001. Wilcoxon signed rank test for the null hypothesis that the median is equal to zero. Cell #1 is shown in **Fig. 7**. Histograms of culture #1 data are shown in **Supplementary Fig. 8**.

Supplementary Note 1

Riesz transform-differential interference contrast microscopy

The Hilbert transform (HT), which shifts the phase of 1D signals by 90°, has been used for calculation of the analytic signal[4]. The HT is defined by a multiplier in the frequency domain:

$$H(u) = -i\text{sign}(u) = -i\frac{u}{|u|}.$$

The HT of a 1D signal $f(x)$ is calculated as follows:

$$F(u) = \mathcal{F}\{f(x)\} = \int_{-\infty}^{\infty} f(x)e^{-2\pi ixu}dx\,,$$

$$F_H(u) = H(u)F(u)\,,$$

$$f_H(x) = \mathcal{F}^{-1}\{F_H(u)\} = \int_{-\infty}^{\infty} F_H(u)e^{2\pi ixu}du\,,$$

where $\mathcal{F}$ and $\mathcal{F}^{-1}$ denote the Fourier transform (FT) and the inverse FT, respectively.

For a 2D signal $f(\mathbf{x})$, the 2D FT $\mathcal{F}_2$ is defined as:

$$F(\mathbf{u}) = \mathcal{F}_2\{f(\mathbf{x})\} = \int_{\mathbb{R}^2} f(\mathbf{x})e^{-2\pi i\mathbf{x}\cdot\mathbf{u}}d\mathbf{x}\,,$$

where $\mathbf{x} = (x, y)^T$ and $\mathbf{u} = (u, v)^T$. Because the HT was originally defined for 1D signals, generalization of the HT to higher dimensions cannot be uniquely defined but includes many approaches[5]. One approach with an introduction of a preferred direction uses a multiplier:

$$H_{\mathbf{n}}(\mathbf{u}) = -i\text{sign}(\mathbf{u}\cdot\mathbf{n})\,,$$

where the direction is denoted by $\mathbf{n} = (\cos\theta, \sin\theta)^T$. When $\mathbf{n}$ is set to the shear axis of a DIC microscope, this directional HT can remove the shadow from DIC images[6] but generates line artifacts (**Fig. 1c**).

The Riesz transform (RT), an extension of the HT to multi-dimensions, is used for generation of the multidimensional analytic signal, the monogenic signal[5]. The RT is also independently and simultaneously proposed as the spiral phase transform[7]. For 2D signals, the RT has two orthogonal components. The multiplier $\mathbf{R}(\mathbf{u})$ is given as follows (**Supplementary Fig. 9a**):

$$\mathbf{R}(\mathbf{u}) = \begin{bmatrix} R_x(\mathbf{u}) \\ R_y(\mathbf{u}) \end{bmatrix} = -i\frac{\mathbf{u}}{\|\mathbf{u}\|} = \frac{-i}{\sqrt{u^2+v^2}}\begin{bmatrix} u \\ v \end{bmatrix}.$$

The RT of a 2D signal $f(\mathbf{x})$ is calculated as:

$$F(\mathbf{u}) = \mathcal{F}_2\{f(\mathbf{x})\},$$

$$\mathbf{F}_R(\mathbf{u}) = \mathbf{R}(\mathbf{u})F(\mathbf{u}),$$

$$\mathbf{f}_R(\mathbf{x}) = \mathcal{F}_2{}^{-1}\{\mathbf{F}_R(\mathbf{u})\}.$$

The directional RT[8] toward $\mathbf{n}$ can be defined by a multiplier:

$$R_{\mathbf{n}}(\mathbf{u}) = \mathbf{R}(\mathbf{u}) \cdot \mathbf{n} = -i\frac{\mathbf{u} \cdot \mathbf{n}}{\|\mathbf{u}\|} = -i\frac{u\cos\theta + v\sin\theta}{\sqrt{u^2 + v^2}}.$$

We demonstrated that the RT directed along the shear axis ($\theta = 45°$) (**Fig. 1d**) effectively removes the shadow-cast appearance of DIC images without generating line artifacts. The second-order RT, which means the RT twice for 180° phase shifting or intensity inversion, can be further defined as:

$$R_{\mathbf{n}}{}^2(\mathbf{u}) = (\mathbf{R}(\mathbf{u}) \cdot \mathbf{n})^2 = -\frac{(\mathbf{u} \cdot \mathbf{n})^2}{\|\mathbf{u}\|^2} = -\frac{(u\cos\theta + v\sin\theta)^2}{u^2 + v^2}.$$

We used this second-order RT directed to the axis perpendicular to the shear ($\theta = 135°$) for selective extraction of absorption information along the shear axis (**Fig. 1e**). For simultaneous conversion of phase information and absorption information, a composite RT filter was constructed by addition of the first-order and second-order RT multipliers (**Fig. 1f**):

$$R_c(\mathbf{u}) = R_{\mathbf{n_1}}(\mathbf{u}) + R_{\mathbf{n_2}}{}^2(\mathbf{u}) = \mathbf{R}(\mathbf{u}) \cdot \mathbf{n_1} + (\mathbf{R}(\mathbf{u}) \cdot \mathbf{n_2})^2$$

$$= -\frac{(u\cos\theta_2 + v\sin\theta_2)^2}{u^2 + v^2} - i\frac{u\cos\theta_1 + v\sin\theta_1}{\sqrt{u^2 + v^2}},$$

where $\mathbf{n_1} = (\cos\theta_1, \sin\theta_1)^T$ and $\mathbf{n_2} = (\cos\theta_2, \sin\theta_2)^T$ denote the direction of the shear axis and its orthogonal direction, respectively.

DIC microscopic images inevitably generate a gradual intensity change in the background that should be removed by flat-field correction[9]. To cut off DC (direct-current) and low-frequency components, the composite RT filter was multiplied by a high-pass filter:

$$HPF(\mathbf{u}) = \begin{cases} 0 & if\ \|\mathbf{u}\|_\infty < u_{cut} \\ 1 & otherwise \end{cases},$$

where u_{cut} is the cut-off frequency. The modified composite multiplier is given by:

$$R_{\mathrm{c}}(\mathbf{u}) = \left[R_{\mathrm{n_1}}(\mathbf{u}) + R_{\mathrm{n_2}}{}^2(\mathbf{u})\right]HPF(\mathbf{u}).$$

This multiplier effectively removed the background shading (compare **Fig. 2e** and **f**; **Supplementary Fig. 2h** and **Fig. 2g**) and was used for RT-DIC imaging.

In summary, a shadow-cast DIC image $f(\mathbf{x})$ can be converted to a self-luminous intensity image $f_R(\mathbf{x})$ by the simple three-step calculation (the 2D FT, multiplication by $R_\mathrm{c}(\mathbf{u})$ (**Supplementary Fig. 9b**) and the inverse 2D FT):

$$F(\mathbf{u}) = \mathcal{F}_2\{f(\mathbf{x})\}\,,$$

$$F_R(\mathbf{u}) = R_\mathrm{c}(\mathbf{u})F(\mathbf{u})\,,$$

$$f_R(\mathbf{x}) = {\mathcal{F}_2}^{-1}\{F_R(\mathbf{u})\}.$$

Supplementary Note 2

Feature estimation with structure tensor analysis

3D intensity image datasets acquired with RT-DIC imaging or fluorescence imaging are analyzed for their geometric features with the structure tensor[10]. For a 3D image $I(\mathbf{x})$, where $\mathbf{x} = (x, y, z)$, smooth image gradients are calculated as:

$$\nabla I = \left(I_x, I_y, I_z\right)^T = G_\mathrm{in} * \left(\frac{\partial I}{\partial x}, \frac{\partial I}{\partial y}, \frac{\partial I}{\partial z}\right)^T ,$$

where G_in is a Gaussian function with a standard deviation σ_in, which is called the inner or noise scale:

$$G_\mathrm{in} = \frac{1}{(2\pi)^{\frac{3}{2}}{\sigma_\mathrm{in}}^3} e^{-\frac{\|\mathbf{x}\|^2}{2{\sigma_\mathrm{in}}^2}} .$$

The local structure tensor is calculated for each voxel:

$$\mathbf{S}(\mathbf{x}) = G_\mathrm{out} * (\nabla I)(\nabla I)^T = G_\mathrm{out} * \begin{bmatrix} {I_x}^2 & I_xI_y & I_xI_z \\ I_xI_y & {I_y}^2 & I_yI_z \\ I_xI_z & I_yI_z & {I_z}^2 \end{bmatrix},$$

where G_out is a Gaussian with the outer or integration scale σ_out, which represents the scale of interest. The tensor is eigendecomposed into eigenvectors $\mathbf{e_1}, \mathbf{e_2}, \mathbf{e_3}$ and eigenvalues $\lambda_1, \lambda_2, \lambda_3$, where $\lambda_1 \geq \lambda_2 \geq \lambda_3 \geq 0$:

$$\mathbf{S} = \lambda_1\mathbf{e_1}{\mathbf{e_1}}^T + \lambda_2\mathbf{e_2}{\mathbf{e_2}}^T + \lambda_3\mathbf{e_3}{\mathbf{e_3}}^T .$$

The tensor can be rewritten according to a protocol modified from the shape classification of the diffusion tensor derived from magnetic resonance imaging[11, 12]:

$$\mathbf{S} = (\lambda_1 + \lambda_2 + \lambda_3)\left(C_p\mathbf{S_p} + C_l\mathbf{S_l} + C_s\mathbf{S_s}\right),$$

where

$$\mathbf{S_p} = \mathbf{e_1}\mathbf{e_1}^T,$$

$$\mathbf{S_l} = \frac{1}{2}(\mathbf{e_1}\mathbf{e_1}^T + \mathbf{e_2}\mathbf{e_2}^T),$$

$$\mathbf{S_s} = \frac{1}{3}(\mathbf{e_1}\mathbf{e_1}^T + \mathbf{e_2}\mathbf{e_2}^T + \mathbf{e_3}\mathbf{e_3}^T),$$

$$C_p = \frac{\lambda_1 - \lambda_2}{\lambda_1 + \lambda_2 + \lambda_3},$$

$$C_l = \frac{2(\lambda_2 - \lambda_3)}{\lambda_1 + \lambda_2 + \lambda_3},$$

$$C_s = \frac{3\lambda_3}{\lambda_1 + \lambda_2 + \lambda_3},$$

$$C_p + C_l + C_s = 1.$$

$\mathbf{S_p}, \mathbf{S_l}, \mathbf{S_s}$ and C_p, C_l, C_s are the planar, linear, and spherical tensor bases and certainty indices, respectively (**Supplementary Fig. 10**). Note that the planar and linear bases in the structure tensor are swapped with those in the diffusion tensor. Note also that the orientation is represented by $\mathbf{e_3}$ in the structure tensor, but by $\mathbf{e_1}$ in the diffusion tensor. The certainty of anisotropy, which represents the certainty of the fiber structure, is defined as:

$$C_a = C_p + C_l = 1 - C_s = \frac{\lambda_1 + \lambda_2 - 2\lambda_3}{\lambda_1 + \lambda_2 + \lambda_3}.$$

Geometrically, $\mathbf{e_3}$ represents the orientation to which the intensity shows the fewest changes, pointing to the orientation of fiber structures. The orientation $\mathbf{e_3}$ is converted to a direction $\mathbf{d}$ by flipping the sign, depending on the relative position from the reference point $\mathbf{x_0}$:

$$\mathbf{d} = \mathrm{sign}\left(\mathbf{e_3} \cdot (\mathbf{x} - \mathbf{x_0})\right)\mathbf{e_3}.$$

In the case of growth cone imaging, the direction of the filopodia extending from the growth cone body is important. The volume of interest is set to cover the filopodia growing upward from the growth cone body at the bottom (**Fig. 2a**). Thus, the reference point $\mathbf{x_0}$ is set to the center of the growth cone body that is usually located beneath the volume of interest (**Supplementary Fig. 11**). In the case of whole cell imaging, the

radial axis is used for conversion from orientation to direction. The reference point $\mathbf{x_0}$ is set to the center of the cell that is defined by the intensity-weighted centroid.

For identification of distal tip structures, the first-order structure tensor or the smoothed gradient is also calculated as:

$$\mathbf{P}(\mathbf{x}) = G_{\text{out}} * \nabla I = G_{\text{out}} * \begin{bmatrix} I_x \\ I_y \\ I_z \end{bmatrix},$$

$$\mathbf{P} = \lambda_p \mathbf{e_p} \,,$$

where $\mathbf{e_p}$ and λ_p represent the polarity unit vector and the magnitude of the gradient, respectively. The distal tip structure is estimated by the anti-parallel relationship between the fiber direction $\mathbf{d}$ and the gradient at the distal endpoint. The certainty index of the distal tip is defined as:

$$C_t = -\left(\mathbf{d} \cdot \frac{\mathbf{P}}{\bar{p}}\right),$$

where $\bar{p}$ denotes the mean norm of $\mathbf{P}$ among the image datasets for normalization of the gradient.

Each voxel in the volume of interest can be tracked backward with the direction $\mathbf{d}$. Voxels that can be sequentially tracked down to the bottom surface of the volume are defined as components of an object that extends from the bottom. This retrograde tracking selectively extracts the filopodia extending upward from the growth cone body, while excluding the collagen fibers that run in random directions.

Supplementary Note 3

Motion estimation with optical flow analysis

a. 3D optical flow calculation with Horn-Schunck method

The optical flow between two image frames can be calculated by solving the motion constraint equation with the Horn-Schunck method[13]. The original method developed for 2D images was modified for 3D images. When a 3D image with intensity $I(x, y, z, t)$ moves by $(\delta x, \delta y, \delta z)$ within time δt, the motion constraint equation is given as:

$$I(x, y, z, t) = I(x + \delta x, y + \delta y, z + \delta z, t + \delta t)\,.$$

Taylor series expansion can rewrite the equation to:

$$I_x u + I_y v + I_z w + I_t = \nabla I \cdot \mathbf{V} + I_t = 0\,,$$

where $\nabla I = \left(I_x, I_y, I_y\right) = \left(\frac{\partial I}{\partial x}, \frac{\partial I}{\partial y}, \frac{\partial I}{\partial z}\right)$ denotes the image gradient, $I_t = \frac{\partial I}{\partial t}$ the partial derivative along time, and $\mathbf{V} = (u, v, w)^T = \left(\frac{\delta x}{\delta t}, \frac{\delta y}{\delta t}, \frac{\delta z}{\delta t}\right)^T$ the image velocity or optical flow. The Horn-Schunck method chooses $\mathbf{V}$ that minimizes the following functional:

$$E[\mathbf{V}] = \iiint_{\mathbb{D}} L dxdydz = \iiint_{\mathbb{D}} \left(L_{data} + \alpha^2 L_{reg}\right) dxdydz\,,$$

$$L_{data} = F^2 = (\nabla I \cdot \mathbf{V} + I_t)^2\,,$$

$$L_{reg} = \|\nabla u\|^2 + \|\nabla v\|^2 + \|\nabla w\|^2\,,$$

where $\mathbb{D}$ is the domain of the 3D image, L_{data} is the data term from the motion constraint equation, L_{reg} is the regularization or smoothness term so that neighboring voxels have a similar velocity, and α is the smoothness constant. The optimal velocity can be calculated by solving the Euler-Lagrange equations. They are rewritten to the three equations:

$$\alpha^2 \Delta u = I_x F\,,$$

$$\alpha^2 \Delta v = I_y F\,,$$

$$\alpha^2 \Delta w = I_z F\,.$$

The Laplacian can be discretized as:

$$\Delta f = f_{xx} + f_{yy} + f_{zz} = L_3 * f = C\left(\bar{f} - f\right) = C(\overline{L_3} * f - f)\,,$$

where L_3 is a discretized 3D Laplacian matrix, $\bar{f}$ is a weighted intensity average of surrounding voxels, $\overline{L_3}$ is the matrix for calculation of the average, and C is a constant determined by the matrix. We use a 26-point stencil for $\overline{L_3}$ with $C = \frac{44}{13}$:

$$\overline{L_3} = \frac{1}{88}\begin{bmatrix} 2 & 3 & 2 & 3 & 6 & 3 & 2 & 3 & 2 \\ 3 & 6 & 3\,; & 6 & 0 & 6\,; & 3 & 6 & 3 \\ 2 & 3 & 2 & 3 & 6 & 3 & 2 & 3 & 2 \end{bmatrix}.$$

The equations can be discretized as:

$$\begin{bmatrix} {I_x}^2 + C\alpha^2 & I_x I_y & I_x I_z \\ I_x I_y & {I_y}^2 + C\alpha^2 & I_y I_z \\ I_x I_z & I_y I_z & {I_z}^2 + C\alpha^2 \end{bmatrix} (\mathbf{V}\text{-}\overline{\mathbf{V}}) = -\bar{F} \begin{bmatrix} I_x \\ I_y \\ I_z \end{bmatrix},$$

where

$$\overline{\mathbf{V}} = (\bar{u}, \bar{v}, \bar{w})^T = \overline{L_3} * \mathbf{V}\,,$$

$$\bar{F} = \nabla I \cdot \bar{\mathbf{V}} + I_t = I_x \bar{u} + I_y \bar{v} + I_z \bar{w} + I_t \,.$$

The solution of the matrix equation is:

$$\mathbf{V} - \bar{\mathbf{V}} = -\frac{\bar{F}}{D} (\nabla I)^T \,,$$

where

$$D = \|\nabla I\|^2 + C\alpha^2 \,.$$

The velocity $\mathbf{V}$ can be calculated with the Jacobi method using the following iterative equation:

$$\mathbf{V}^{k+1} = \bar{\mathbf{V}}^k - \frac{\bar{F}^k}{D} (\nabla I)^T \,,$$

where

$$\bar{\mathbf{V}}^k = \overline{L_3} * \mathbf{V}^k \,,$$

$$\bar{F}^k = \nabla I \cdot \bar{\mathbf{V}}^k + I_t \,.$$

For over-relaxation with a relaxation factor ω (≈ 1.5), we use the equation:

$$\mathbf{V}^{k+1} = (1-\omega)\mathbf{V}^k + \omega \left[\bar{\mathbf{V}}^k - \frac{\bar{F}^k}{D} (\nabla I)^T \right] .$$

b. Multi-scale optical flow calculation with pyramidal implementation

The Horn-Schunck method cannot essentially detect an optical flow that is larger than the voxel size. Thus, we utilize the coarse-to-fine multi-scale strategy with Gaussian pyramids and image warping[14] . **Supplementary Fig. 12** shows pyramidal implementation of the optical flow calculation. We constructed Gaussian pyramids of the reference and test image frames by smoothing with a Gaussian filter and two-fold downsampling. The velocity was calculated with the 3D Horn-Schunck optical flow method at the top level of the pyramids. The velocity was expanded by two-fold multiplication, two-fold upsampling and smoothing by interpolation to estimate the coarse initial velocity $\mathbf{V_i}$ at the lower level of the pyramids. After moving to the lower level, the images of the test frames were warped with the expanded velocity vectors. The fine residual velocity $\mathbf{V_r}$ was calculated similarly between the current images and the warped residual images. The summed total velocity at this level is calculated as $\mathbf{V} = \mathbf{V_i} + \mathbf{V_r}$. These steps were repeated until level zero where the original images are

located. Thus, we finally obtained the correctly estimated velocity. Practically, we constructed level 5 pyramids from 256 × 256 × 160 voxel images for detection of displacements as large as 2 μm.

c. Estimation of translational and rotational kinematics

For each voxel at $\mathbf{x} = (x, y, z)$ and time t, we calculated four sets of image velocity vectors with the multi-scale optical flow analysis (**Supplementary Fig. 13a**). These consist of single-frame forward velocity $\mathbf{Vf}$ to time $t + \delta t$, single-frame backward velocity $\mathbf{Vb}$ from $t - \delta t$, double-frame forward velocity $\mathbf{Vff}$ to time $t + 2\delta t$, and double-frame backward velocity $\mathbf{Vbb}$ from time $t - 2\delta t$.

Translational physical quantities including velocity, acceleration and jerk are derived from these four vectors. The next velocity $\mathbf{Vn}$, the previous velocity $\mathbf{Vp}$, and the central velocity $\mathbf{Vc}$ are defined as:

$$\mathbf{Vn}(\mathbf{x}, t) = \mathbf{Vff}(\mathbf{x}, t) - \mathbf{Vf}(\mathbf{x}, t)\,,$$

$$\mathbf{Vp}(\mathbf{x}, t) = \mathbf{Vb}(\mathbf{x}, t) - \mathbf{Vbb}(\mathbf{x}, t)\,,$$

$$\mathbf{Vc}(\mathbf{x}, t) = \frac{\mathbf{Vb}(\mathbf{x}, t) + \mathbf{Vf}(\mathbf{x}, t)}{2}\,.$$

The acceleration vector and the jerk vector are calculated as:

$$\mathbf{A}(\mathbf{x}, t) = \frac{-\mathbf{Vn} + 15\mathbf{Vf} - 15\mathbf{Vb} + \mathbf{Vp}}{12\delta t},$$

$$\mathbf{J}(\mathbf{x}, t) = \frac{\mathbf{Vn} - \mathbf{Vf} - \mathbf{Vb} + \mathbf{Vp}}{2\delta t^2}.$$

Rotational physical quantities are estimated by calculation of the Frenet-Serret TNB (tangent-normal-binormal) frames (**Supplementary Fig. 13b**).

Tangent vectors are given by:

$$\mathbf{eTp} = \frac{\mathbf{Vp}}{||\mathbf{Vp}||}\,,$$

$$\mathbf{eTb} = \frac{\mathbf{Vb}}{||\mathbf{Vb}||}\,,$$

$$\mathbf{eTf} = \frac{\mathbf{Vf}}{||\mathbf{Vf}||}\,,$$

$$\mathbf{eTn} = \frac{\mathbf{Vn}}{\|\mathbf{Vn}\|} \ .$$

Binormal vectors are given by:

$$\mathbf{eBp} = \frac{\mathbf{eTp} \times \mathbf{eTb}}{\|\mathbf{eTp} \times \mathbf{eTb}\|},$$

$$\mathbf{eB} = \frac{\mathbf{eTb} \times \mathbf{eTf}}{\|\mathbf{eTb} \times \mathbf{eTf}\|},$$

$$\mathbf{eBn} = \frac{\mathbf{eTf} \times \mathbf{eTn}}{\|\mathbf{eTf} \times \mathbf{eTn}\|}.$$

Rotational motion in 3D space consists of a curvature component defined as the rotation of the tangent vector about the binormal vector, and a torsional component defined as the rotation of the binormal vector about the tangent vector. The curvature angular velocity vector is given by:

$$\boldsymbol{\omega}_{\mathbf{c}} = \frac{1}{\delta t} \mathrm{atan2}(\|\mathbf{eTb} \times \mathbf{eTf}\|, \mathbf{eTb} \cdot \mathbf{eTf})\ \mathbf{eB}\,,$$

where $\mathrm{atan2}(y, x)$ is a four-quadrant arctangent function of y/x. The torsional angular velocity vector is given by:

$$\boldsymbol{\omega}_{\mathbf{t}} = \frac{\boldsymbol{\omega}_{\mathbf{tb}} + \boldsymbol{\omega}_{\mathbf{tf}}}{2} \ ,$$

where

$$\boldsymbol{\omega}_{\mathbf{tb}} = \frac{1}{\delta t} \mathrm{atan2}(\|\mathbf{eBp} \times \mathbf{eB}\|, \mathbf{eBp} \cdot \mathbf{eB})\mathbf{eTb} \ ,$$

$$\boldsymbol{\omega}_{\mathbf{tf}} = \frac{1}{\delta t} \mathrm{atan2}(\|\mathbf{eB} \times \mathbf{eBn}\|, \mathbf{eB} \cdot \mathbf{eBn})\mathbf{eTf} \ .$$

The total angular velocity vector is calculated as:

$$\boldsymbol{\omega} = \boldsymbol{\omega}_{\mathbf{c}} + \boldsymbol{\omega}_{\mathbf{t}} \ .$$

Supplementary References

1. Arnison MR, Larkin KG, Sheppard CJ, Smith NI, Cogswell CJ. Linear phase imaging using differential interference contrast microscopy. *J Microsc* **214**, 7-12 (2004).

2. Larkin KG, Fletcher PA. Isotropic scalar image visualization of vector differential image data using the inverse Riesz transform. *Biomedical optics express* **5**, 907-920 (2014).

3. Shribak M, Larkin KG, Biggs D. Mapping optical path length and image enhancement using quantitative orientation-independent differential interference contrast microscopy. *J Biomed Opt* **22**, 16006 (2017).

4. Gabor D. Theory of communication. Part 1: The analysis of information. *Journal of the Institution of Electrical Engineers - Part III: Radio and Communication Engineering* **93**, 429-441 (1946).

5. Felsberg M, Sommer G. The monogenic signal. *IEEE Transactions on Signal Processing* **49**, 3136-3144 (2001).

6. Arnison MR, Cogswell CJ, Smith NI, Fekete PW, Larkin KG. Using the Hilbert transform for 3D visualization of differential interference contrast microscope images. *J Microsc* **199**, 79-84 (2000).

7. Larkin KG, Bone DJ, Oldfield MA. Natural demodulation of two-dimensional fringe patterns. I. General background of the spiral phase quadrature transform. *J Opt Soc Am A Opt Image Sci Vis* **18**, 1862-1870 (2001).

8. Unser M, Ville DVD. Higher-order riesz transforms and steerablewavelet frames. In: *2009 16th IEEE International Conference on Image Processing (ICIP)* (ed^(eds) (2009).

9. Murphy DB, Davidson MW. *Fundamentals of light microscopy and electronic imaging*, 2nd edn. Wiley-Blackwell (2013).

10. Bigün J, Granlund G. Optimal orientation detection of linear symmetry. In: *IEEE First International Conference on Computer Vision* (ed^(eds) (1987).

11. Westin CF, Maier SE, Mamata H, Nabavi A, Jolesz FA, Kikinis R. Processing and visualization for diffusion tensor MRI. *Med Image Anal* **6**, 93-108 (2002).

12. Westin CF, Peled S, Gudbjartsson H, Kikinis R, Jolesz FA. Geometrical diffusion measures for MRI from tensor basis analysis. In: *International Society for Magnetic Resonance in Medicine (ISMRM) '97* (ed^(eds) (1997).

13. Horn BK, Schunck BG. Determining optical flow. *Artificial Intelligence* **17**, 185-203 (1981).

14. Burt PJ, Adelson EH. The Laplacian pyramid as a compact image code. *IEEE Transactions on Communications* **31**, 532-540 (1983).
