# Supplementary material for: Revealing chiral cell motility by 3D Riesz transform-differential interference contrast microscopy and computational kinematic analysis
Source: Nat Commun. 2017 Dec 19;8:2194. doi: 10.1038/s41467-017-02193-w (PMC5736583; doi:10.1038/s41467-017-02193-w)
Supplement: Supplementary file 3 — Descriptions of Additional Supplementary Files [file 41467_2017_2193_MOESM3_ESM.pdf]

Descriptions of Additional Supplementary Files

File Name: Supplementary Dataset 1
Description: A zip file of image data for neuronal growth cone, neurite growth and *Dictyostelium* cell migration. These are part of data used in this study and can also be used as sample data for demonstration of **Supplementary Software 2-4**.

File Name: Supplementary Movie 1
Description: Time-lapse movie of a GFP-labeled growth cone on 2D surface with sequential RT-DIC and fluorescence imaging. RT-DIC imaging for 300 sec (gray for DIC, magenta for RT-DIC), interval for 20 sec, and then GFP imaging for 300 sec (green). 30 frame per s, 30× actual speed.

File Name: Supplementary Movie 2
Description: Z-scan image series of a growth cone. Cell #2. Same frame as **Fig. 2, 3 and 4**. Images are shown from the top to the bottom and in the order of raw DIC (gray), thresholded DIC (red and blue for positive negative slopes, respectively), positive RT-DIC (green) on thresholded DIC, positive RT-DIC, and positive RT-DIC on raw DIC.

File Name: Supplementary Movie 3
Description: Time-lapse movie of 3D rendering of a growth cone. Top and backside views. Cell #2. 30 frame per s, 30× actual speed.

File Name: Supplementary Movie 4
Description: Time-lapse movie showing 3D structure of a growth cone. Fiber (blue) and tip (red) structures judged from the certainties are shown on the RT-DIC intensity (green). Top and backside views. Cell #2. 30 frame per s, 30× actual speed.

File Name: Supplementary Movie 5
Description: Time-lapse movie showing 3D motility of a growth cone. Magnitude of velocity (blue) are overlaid on RT-DIC image (green). Top view. Cell #2. 30 frame per s, 30× actual speed.

File Name: Supplementary Movie 6
Description: Time-lapse movie showing axial motility of a growth cone. Left panel: Distribution of local axial velocity. Overlay of extensive (positive, red) and retractive (negative, blue) axial velocity on RT-DIC image (green). Right panel: Distribution of local axial angular velocity. Overlay of right-screw (positive, red) and left-screw (negative, blue) axial angular velocity on RT-DIC image (green). Backside views. Cell #2. 10 frame per s, 10× actual speed.

File Name: Supplementary Movie 7
Description: Time-lapse movie showing trajectories of particles within a growth cone. Tractable voxels are marked with six colors. The voxels that appeared at the same frame are shown with the same color. Backside views. Cell #2. 10 frame per s, 10× actual speed.

File Name: Supplementary Movie 8
Description: Time-lapse images of Dictyostelium cells on 2D glass surface. Single-position, short-term, high-density culture #1. 3-4 hours after starvation. Movies are sequentially presented in the order of raw DIC image (gray), velocity magnitude (green) and angular velocity (red for CW, blue for CCW rotation). 30 frame per s, 300× actual speed.

File Name: Supplementary Movie 9

Description: Time-lapse images showing segmentation and tracking of Dictyostelium cells on 2D glass surface. Multi-position, long-term, low-density culture #1. Cropped images during 7-8 hours after starvation. Raw DIC images (gray) and cell particles segmented by the smoothed RT-DIC intensity (gray ovals) are overlaid by the optical flow-tracked cell centroids (colored squares). 10 frame per s, 300× actual speed.

File Name: Supplementary Movie 10
Description: Time-lapse images showing trajectories of migrating *Dictyostelium* cells on 2D glass surface. Multi-position, long-term, low-density culture #1. Full images (3 mm × 3 mm area) during 3-12 hours after starvation. Positions of tractable cells are color coded with tails for 20 min. 30 frame per s, 900× actual speed.

File Name: Supplementary Movie 11
Description: Whole cell structure of *Dictyostelium* in 3D collagen gels. Intensity-weighted centroid (blue) and tip structure (magenta) were overlaid on RT-DIC images (green). Rotated view. Cell #1.

File Name: Supplementary Movie 12
Description: Time-lapse movie showing 3D Motility of *Dictyostelium* in collagen gels. Top and backside views. Intensity-weighted centroid (blue) and tip structure (magenta) were overlaid on RT-DIC images (green). Cell #1. 10 frame per s, 40× actual speed.

File Name: Supplementary Software 1
Description: A zip file of an ImageJ plugin and its source code for RT-DIC conversion.

File Name: Supplementary Software 2
Description: A zip file of MATLAB codes for RT-DIC conversion and image processing.

File Name: Supplementary Software 3
Description: A zip file of MATLAB codes for structure analysis.

File Name: Supplementary Software 4
Description: A zip file of MATLAB codes for motion analysis.
