# Supplementary material for: Revealing chiral cell motility by 3D Riesz transform-differential interference contrast microscopy and computational kinematic analysis
Source: Nat Commun. 2017 Dec 19;8:2194. doi: 10.1038/s41467-017-02193-w (PMC5736583; doi:10.1038/s41467-017-02193-w)
Supplement: Supplementary file 17 — Supplementary Software 1 [file 41467_2017_2193_MOESM17_ESM.zip › READMESoftware1.pdf]

Lumino DIC
ver.1.0.0

"**Lumino DIC**" is an ImageJ plugin that converts a shadow-cast differential interference contrast (DIC) image to a shadow-free self-luminous image by the composite Riesz transform (RT). This conversion enables 3D visualization and intensity-based analysis of DIC images that are acquired with a conventional DIC microscope. See the following literature for the detailed methods.

Reference
Tamada A and Igarashi M (2017)
Revealing chiral cell motility revealed by computational kinematic analysis with 3D Riesz transform-differential interference contrast microscopy and computational kinematic analysis.
Nature Communications in press.

Requirements
ImageJ or Fiji with Java Runtime Environment (JRE)

Installation
Download and unzip this package.
Copy the jar file "Lumino_DIC-1.0.0.jar" to "plugins" folder of ImageJ or Fiji.
Restart ImageJ/Fiji or run "Refresh Menus" command from "Help" menu.

How to use
1. Open a DIC image or image stack to be converted.
2. Run "Lumino DIC" from "Plugins" menu.
3. Set the following parameters in the dialog:

   Shear angle (direction of shadow):

   Set the shear angle of DIC prism by the degrees from the X-axis, so that it points to the direction of shadow. If the shadow appears in bottom-right direction, set the value to 45. If the shadow appears in top-left direction, set it to 225.

   Cut off for flat-field correction:

   This parameter sets the threshold of the high-pass filter for flat-field correction. If the intensity gradient in the background becomes a problem, increase this value from zero. However, too large value would cause loss of information and generate an artifactual image.

   Phase gradient coeff.:

   This sets the coefficient for selective extraction of phase gradient information along the shear axis. The default value is 1.

   Absorption coeff.:

   This sets the coefficient for selective inversion of absorption information along the axis perpendicular to the shear. The default value is 1. Since the absorption

component is usually weaker than the phase gradient component in DIC images, larger value (e.g. 2) may be used for the compensation.

Display composite Riesz mask:

If this checkbox is “ON”, the Fourier filter used for the composite RT will be displayed in a new image stack. The stack shows the real and imaginary components of the filter.

Thresholding (positive intensity only):

If this checkbox is “OFF”, the converted RT-DIC images have both positive and negative intensities around the background with zero values. This option is suitable for a single focal plane image and for comparison with the source DIC image.

If this checkbox is “ON”, the RT-DIC images are thresholded at zero and their negative intensity is discarded. The thresholded RT-DIC images are comparable to fluorescence images. This option is suitable for 3D reconstruction of stack images.

4. Press “OK” and the RT-DIC conversion will be started.
5. The converted image or image stack will appear in a new window with 32-bit float values. The images can be processed or analyzed in the same way as fluorescence images. The thresholded 3D RT-DIC image stack can be immediately reconstructed and visualized with appropriate 3D viewers.

Instead of using the dialog, the RT-DIC conversion can also be executed by a macro with setting of parameters like:
run("Lumino DIC", "shear=45 cut=0 phase=1 absorption=1");
or
run("Lumino DIC", "shear=45 cut=0 phase=1 absorption=1 display thresholding");

Sample images

This package contains sample DIC images that are used in the literature (Tamada and Igarashi, 2017). These DIC images, with a 45-degree shear angle, can be converted to shadow-free bright images by the RT-DIC conversion. The images are stored in the following tiff files:

radialGrating.tif
A synthetic DIC image (**Fig.1** and **Supplementary Fig.1**)

growthCone2D.tif
A DIC image of a neuronal growth cone (**Fig.2** and **Supplementary Fig.2**)

growthCone3D.tif
A 3D image stack of a neuronal growth cone (**Fig.2** and **Fig.3**)

neurite2D.tif
A montage of multiple DIC images showing the neurite growth from an aggregate of neurons (**Fig.5**)

dicty2D.tif
A DIC image showing the migrating cellular slime mold *Dictyostelium discoideum* (**Fig.6**)

Source code
For the source code, see “Lumino_DIC-1.0.0.java” in the package.

License
This software is distributed under the MIT License; see LICENSE.txt.
