# Supplementary figures and images for: Revealing chiral cell motility by 3D Riesz transform-differential interference contrast microscopy and computational kinematic analysis
Source: Nat Commun. 2017 Dec 19;8:2194. doi: 10.1038/s41467-017-02193-w (PMC5736583; doi:10.1038/s41467-017-02193-w)

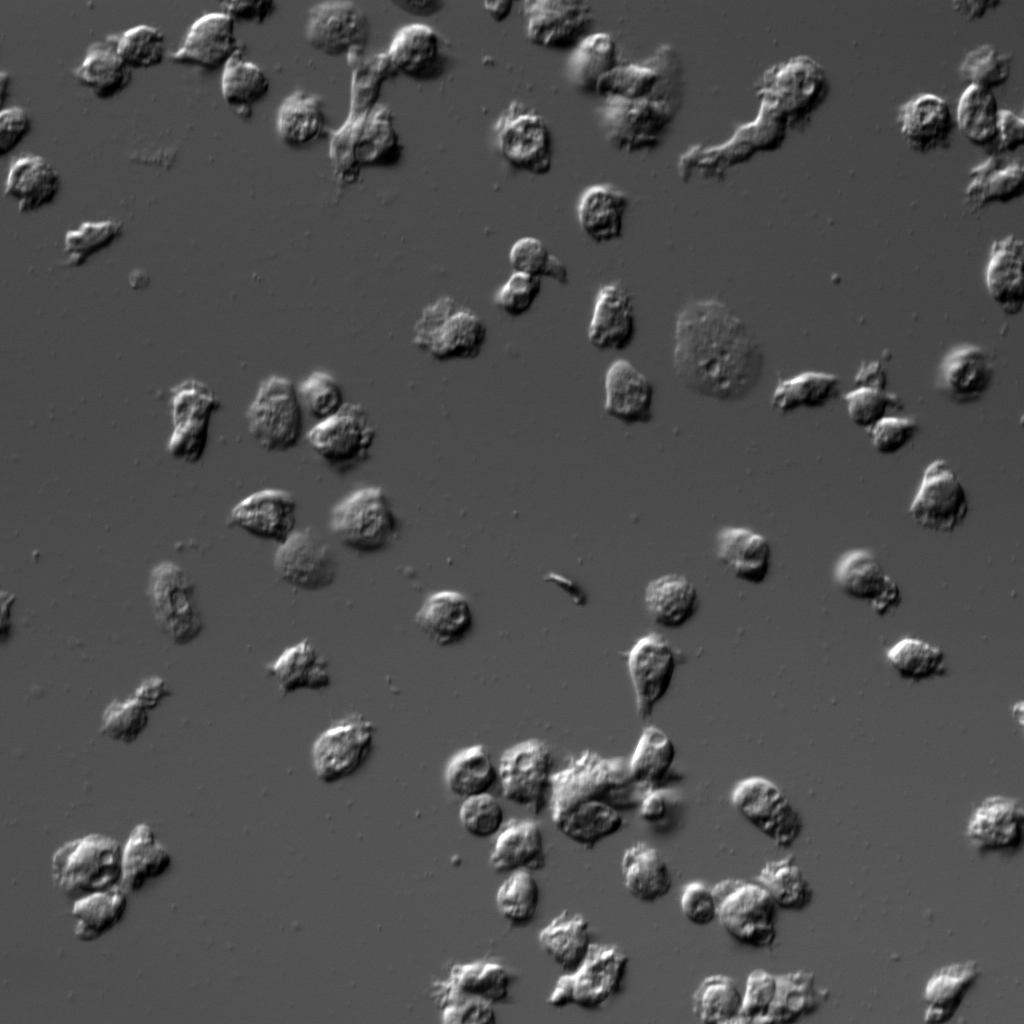

Supplement: Supplementary file 17 — Supplementary Software 1 [file 41467_2017_2193_MOESM17_ESM.zip › dicty2D.tif]

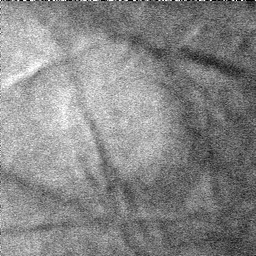

Supplement: Supplementary file 17 — Supplementary Software 1 [file 41467_2017_2193_MOESM17_ESM.zip › growthCone3D.tif]

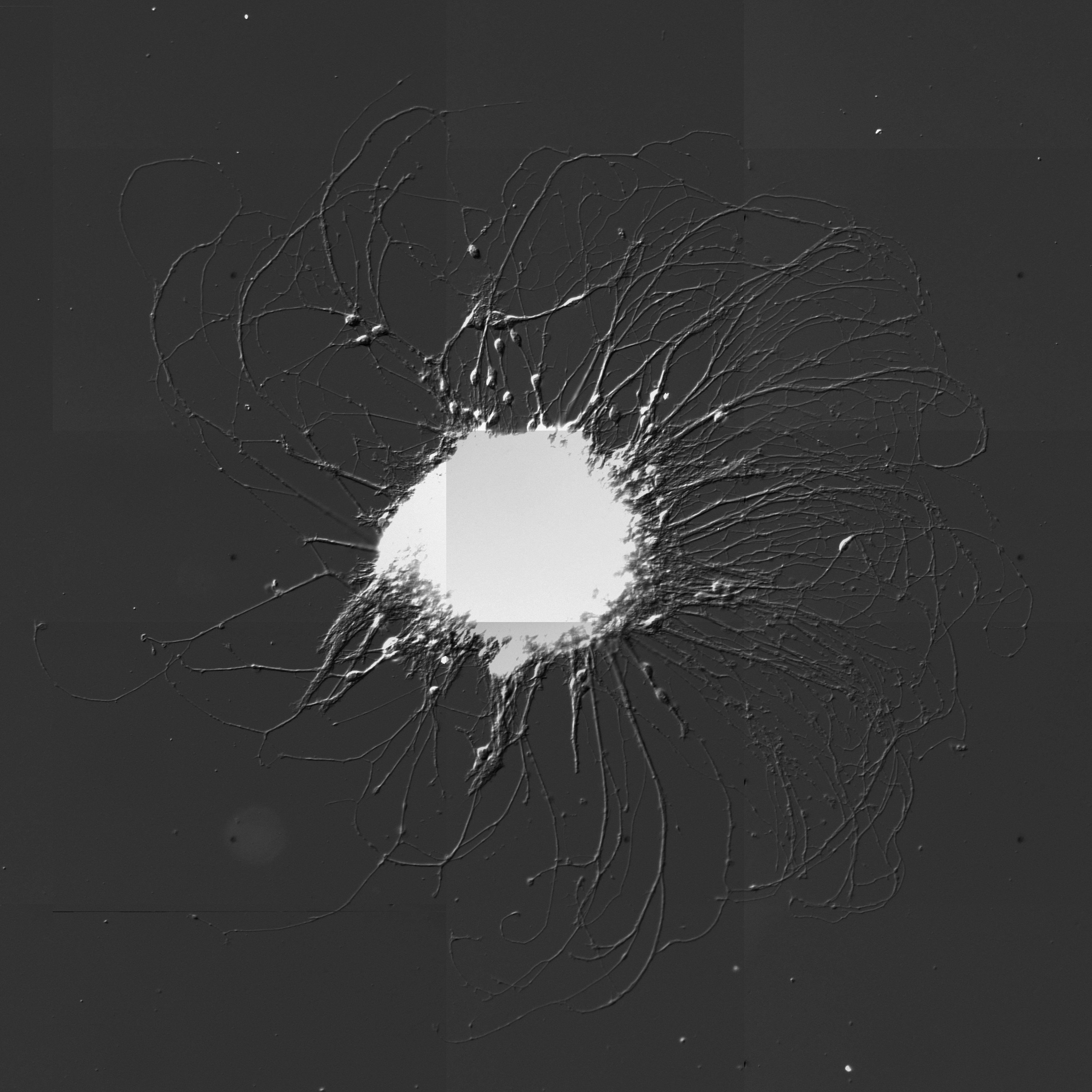

Supplement: Supplementary file 17 — Supplementary Software 1 [file 41467_2017_2193_MOESM17_ESM.zip › neurite2D.tif]

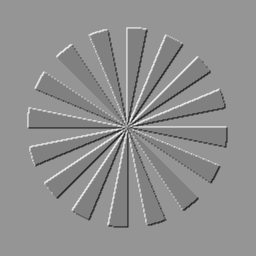

Supplement: Supplementary file 17 — Supplementary Software 1 [file 41467_2017_2193_MOESM17_ESM.zip › radialGrating.tif]
