# Supplementary material for: Revealing chiral cell motility by 3D Riesz transform-differential interference contrast microscopy and computational kinematic analysis
Source: Nat Commun. 2017 Dec 19;8:2194. doi: 10.1038/s41467-017-02193-w (PMC5736583; doi:10.1038/s41467-017-02193-w)
Supplement: Supplementary file 18 — Supplementary Software 2 [file 41467_2017_2193_MOESM18_ESM.zip › READMESoftware2.pdf]

MATLAB codes for Riesz transform and related image processing

These are MATLAB codes to perform image processing for Riesz transform-Differential Interference Contrast (RT-DIC) microscopy and fluorescence microscopy.

Reference

Tamada A and Igarashi M (2017)
Revealing chiral cell motility revealed by computational kinematic analysis with 3D Riesz transform-differential interference contrast microscopy and computational kinematic analysis.
Nature Communications in press.

Requirements

MATLAB software (we confirmed ver. R2013b and R2016b) with parallel computing, image processing, signal processing and statics toolboxes. At least about 10GB memory is required. Adjust the number of parallel pools in the scripts for your environment.

Installation

Download and unzip this package. For demonstration with sample images, unzip **Supplementary Data** at the same level. Then move to folder "**CodeRT**".

How to use

1. **CompositeRieszTransform.m**
   A function to convert a DIC image to a self-luminous image from a workspace variable.
   Execute script "**DemoCompositeRieszTransform.m**" for demonstration with sample images in folder "**DataRT**",
   These data correspond to **Fig. 1**, and **Supplementary Fig. 1.**

2. **ProcessRTDIC3DT.m**
   A function to perform preprocessing, Riesz transform and postprocessing with 3D time-lapse DIC images.
   Execute script "**DemoProcessRieszTransformGrowthCone3D.m**" for demonstration with 3D growth cone images in "**DataGrowthCone3D**".
   These data correspond to **Fig. 2-4** and **Supplementary Fig. 2.**

   Execute script "**DemoProcessRieszTransformDicty3D.m**" for demonstration with 3D Dictyostelium images in "**DataDicty3D**".
   These data correspond to **Fig. 7.**

3. **ProcessFluor3DT.m**
   A function to perform preprocessing and postprocessing with 3D time-lapse fluorescence images.
   No images for demonstration.

4. **ProcessRTDIC2DT.m**

A function to perform preprocessing, Riesz transform and postprocessing with 2D time-lapse DIC images.
Execute script “**DemoProcessRieszTransformDicty2D.m**” for demonstration with 2D Dictyostelium cell migration images in “**DataDicty2D**”.
These data correspond to **Fig. 6.**

5. **ProcessFluor2DT.m**
   A function to perform preprocessing and postprocessing with 2D time-lapse fluorescence images.
   No images for demonstration.

6. **ProcessRTDIC2DS.m**
   A function to perform preprocessing, Riesz transform and postprocessing with 2D still DIC image.
   Execute script “**DemoProcessRieszTransformNeurite2D.m**” for demonstration with 2D neurite growth images in “**DataNeurite2D**”.
   These data correspond to **Fig. 5.**

License

This software is distributed under the MIT License; see LICENSE.txt.
