# Supplementary material for: Revealing chiral cell motility by 3D Riesz transform-differential interference contrast microscopy and computational kinematic analysis
Source: Nat Commun. 2017 Dec 19;8:2194. doi: 10.1038/s41467-017-02193-w (PMC5736583; doi:10.1038/s41467-017-02193-w)
Supplement: Supplementary file 19 — Supplementary Software 3 [file 41467_2017_2193_MOESM19_ESM.zip › READMESoftware3.pdf]

MATLAB codes for structure analysis

These are MATLAB codes to perform structure analysis for multidimensional images with structure tensor.

Reference

Tamada A and Igarashi M (2017)
Revealing chiral cell motility revealed by computational kinematic analysis with 3D Riesz transform-differential interference contrast microscopy and computational kinematic analysis.
Nature Communications in press.

Requirements

MATLAB software (we confirmed ver. R2013b and R2016b) with parallel computing, image processing, signal processing and statics toolboxes. At least about 10GB memory is required. Adjust the number of parallel pools in the scripts for your environment.

Installation

Download and unzip this package. For demonstration with sample images, unzip **Supplementary Data** at the same level. Then move to folder "**CodeStructure**".

How to use

1. **StructureFiber3DT.m**
   A function to analyze fiber structure of 3D time-lapse images.
   Execute script "**DemoStructureGrowthCone3D.m**" for demonstration with 3D growth cone RT-DIC images in "**DataGrowthCone3D**", after RT-DIC conversion by "**DemoProcessRieszTransformGrowthCone3D.m**" in **Supplementary Software2**.
   These data correspond to **Fig. 3-4.**

2. **StructureCell3DT.m**
   A function to analyze cell structure of 3D time-lapse images.
   Execute script "**DemoStructureDicty3D.m**" for demonstration with 3D Dictyostelium RT-DIC images in "**DataDicty3D**", after RT-DIC conversion by "**DemoProcessRieszTransformDicty3D.m**" in **Supplementary Software2**.
   These data correspond to **Fig. 7.**

3. **Structure2DS.m**
   A function to analyze structure of 2D still image.
   Execute script "**DemoStructureNeurite2D.m**" for demonstration with 2D neurite growth RT-DIC images in "**DataNeurite2D**", after RT-DIC conversion by "**DemoProcessRieszTransformNeurite2D.m**" in **Supplementary Software2**.
   These data correspond to **Fig. 5.**

License

This software is distributed under the MIT License; see LICENSE.txt.
