# Supplementary material for: Revealing chiral cell motility by 3D Riesz transform-differential interference contrast microscopy and computational kinematic analysis
Source: Nat Commun. 2017 Dec 19;8:2194. doi: 10.1038/s41467-017-02193-w (PMC5736583; doi:10.1038/s41467-017-02193-w)
Supplement: Supplementary file 20 — Supplementary Software 4 [file 41467_2017_2193_MOESM20_ESM.zip › READMESoftware4.pdf]

MATLAB codes for motion analysis

These are MATLAB codes to perform motion analysis for multidimensional images with Horn-Schunck optical flow and calculation of physical quantities.

Reference
Tamada A and Igarashi M (2017)
Revealing chiral cell motility revealed by computational kinematic analysis with 3D Riesz transform-differential interference contrast microscopy and computational kinematic analysis.
Nature Communications in press.

Requirements
MATLAB software (we confirmed ver. R2013b and R2016b) with parallel computing, image processing, signal processing and statics toolboxes. At least about 10GB memory is required. Adjust the number of parallel pools in the scripts for your environment.

Installation
Download and unzip this package. For demonstration with sample images, unzip **Supplementary Data** at the same level. Then move to folder "**CodeMotion**".

How to use

1. **MotionFiber3D.m**
   A function to analyze motion of fiber structure from 3D time-lapse images.
   Execute script "**DemoMotionGrowthCone3D.m**" for demonstration with 3D growth cone images in "**DataGrowthCone3D**", after structure analysis with "**DemoStructureGrowthCone3D.m**" in **Supplementary Software 3**.
   These data correspond to **Fig. 4** and **Supplementary Fig. 6.**

2. **MotionCell3D.m**
   A function to analyze motion of cell structure from 3D time-lapse images.
   Execute script "**DemoMotionDicty3D.m**" for demonstration with 3D Dictyostelium images in "**DataDicty3D**", structure analysis with "**DemoStructureDicty3D.m**" in **Supplementary Software 3**.
   These data correspond to **Fig. 7** and **Supplementary Fig. 8.**

3. **Motion2D.m**
   A function to analyze motion of 2D time-lapse images.
   Execute script "**DemoMotionDicty2D.m**" for demonstration with 2D Dictyostelium images in "**DataDicty2D**", after RT-DIC conversion by "**DemoProcessRieszTransformDicty2D.m**" in **Supplementary Software 2**.
   These data correspond to **Fig. 6** and **Supplementary Fig. 7.**

License
This software is distributed under the MIT License; see LICENSE.txt.
