# Supplementary material for: Revealing chiral cell motility by 3D Riesz transform-differential interference contrast microscopy and computational kinematic analysis
Source: Nat Commun. 2017 Dec 19;8:2194. doi: 10.1038/s41467-017-02193-w (PMC5736583; doi:10.1038/s41467-017-02193-w)
Supplement: Supplementary file 2 — Peer Review File [file 41467_2017_2193_MOESM2_ESM.pdf]

PEER REVIEW FILE

Reviewers' comments:

Reviewer #1 (Remarks to the Author):

In this paper, the authors present new applications of existing image analysis and signal processing techniques. In particular, they apply these techniques to determine whether cells display chiral biases.
I appreciate the topic and the goal of the paper. However, the article is difficult to follow and the figure are not so convincing.
Furthermore, the number of movies is overwhelming and they are not helpful.
I would urge the authors to make their study more readable and to display the results in a clearer and more intuitive fashion.

Some specific comments:
Page 8: "By visual observation, individual filopodia not only twirled in the direction of a right-handed screw as previously reported but also appeared to exhibit axial spinning. They seemed to show retrograde motion toward the growth cone body, consistent with the retrograde actin flow."
I simply don't see this from the figure or the movies.
Fig. 4d is supposed to show us that right-screw rotation is dominant over left-screw.
I don't quite see that; there are too many colors in the figure.
Also, the quantification of this is in Fig 4g.
Presumably, the values next to the distribution represent the average and SD. The average is slightly positive which, I guess, means right-screw dominance.
This is not explained. Furthermore, the average is very close to zero, can we really draw any conclusions from this? In other words, do we know that this distribution is skewed in a statistically significant way?
Why are the numbers of Fig. 4e and table 4i different? Do they represent different velocity magnitudes?
Fig. 5: the velocity distribution is more skewed than the one in Fig. 4 but, again, how do we know that this skewness is statistically significant?
Fig. 6: Please show a quantitative analysis of the cell trajectories, including the biased clockwise motion. Also, how do cell-cell collision affect this bias?
Furthermore, how is the non-zero rotational motion along an axis perpendicular to the surface going to result curved trajectories? A more mechanistic insight

will be helpful.
Fig. 7: This seems to show that a Dictyostelium cell has many tips.
Normally, in 2D motion, it is believed that these cells have only 1-3 pseudopods.
See, for example, Bosgraaf and van Haastert, Plos One, 2009.
Please comment. Also, how is a tip defined and how is the radial axis defined?
I also have a conceptual difficulty with this figure. It seems to show a right-screw rotation around the axis of motility. Fig. 6 seems to show a right-screw rotation perpendicular to the axis of motility. How are these two related?
Ref. 45 is not relevant to the clockwise or anti-clockwise motion of Dictyostelium aggregates. A better one is Rappel et al, Phys. Rev. Lett. 1999.

Reviewer #2 (Remarks to the Author):

My review will concentrate on the microscopy aspects of the manuscript. This manuscript proposes a composite Riesz transform (RT) operator to visualise and analyse single-shot DIC images. DIC microscopy produces images which are a non-linear mixture of the object amplitude and phase gradient. For transparent specimens with varying refractive index, the phase gradient enables imaging of changes in optical path length, but the resulting images have a shadow cast appearance which complicates the application of visualisation and analysis methods that are often designed for brightness images rather than gradient images.

The composite RT operator proposed in this manuscript is used to convert the shadow cast appearance to a more conventional brightness signal, which can then be thresholded for 3D volume visualisation and analysed using computer vision algorithms. The computer vision analysis has been used to produce comprehensive statistics of the left-right asymmetry of motion and growth in multiple samples of multiple organisms.

The combination and adaptation of methods from multiple disciplines is impressive and well described, and offers an insight into the origins and mechanisms of left-right asymmetry. A key advance in this manuscript is the introduction of a composite RT which enables the use of single-shot DIC capture to collect and subsequently analyse structural information. Single-shot methods are highly valuable for rapid capture of living 3D cells. I think the paper has the potential to be highly influential in demonstrating the application of advanced microscopy and image processing methods to a compelling biomedical problem.

The authors are careful to introduce the topics of image processing of DIC images and, separately, the Riesz transform. However, there is a significant history of application of the Riesz transform specifically to DIC images, which is currently not referenced at all:
• Applying the inverse Riesz transform to images captured using an optical variant of DIC was

first proposed in (Arnison, 2004) where it was used to integrate the phase gradient to estimate the linear phase.

• A recent paper by (Shribak, 2017) proposes using the inverse Riesz transform for visualisation of images captured using another optical variant of DIC. This paper also serves as a brief review of the last 13 years of literature on Riesz transforms and DIC.

Although I have listed papers which use the inverse RT, the forward and inverse RT operations are closely related.

These papers all rely on multi-shot variants of DIC, so the use of the RT with single-shot DIC in the manuscript remains novel. The claims on page 14 that the proposed method can be used with standard a DIC microscope are justified.

An additional issue is that while the manuscript correctly cites (Felsberg, 2001), the Riesz transform as a 2D extension of the 1D Hilbert transform was independently and simultaneously proposed by (Larkin, 2001). The manuscript specifically relies on a directional RT, which was introduced by (Unser, 2009).

Given the importance of the RT to the methods of the paper, and the weight being placed on the novelty of the RT being used in this context, I would recommend revisions be made to clarify the relationship between the composite RT method proposed in the manuscript, and the literature on RT and DIC.

I also have some minor comments and suggestions:

1. Page 19, Line 402: "the intensity was expanded to a full 12-bit range." What does expanded mean? The camera captured data at 12-bit, so working in 12-bits during processing is merely maintaining, not expanding, the range. In addition, I would expect that the Fourier processing described would likely be performed in MATLAB in single precision float (32-bit).
2. Page 20, lines 413-414. The images were "often" deconvolved. Please clarify "often", as this is overly vague. Deconvolution requires a calibrated or estimated PSF. How was the PSF determined?

References

Arnison, Matthew R., Kieran G. Larkin, Colin J. R. Sheppard, Nicholas I. Smith, and Carol J. Cogswell. 2004. “Linear Phase Imaging Using Differential Interference Contrast Microscopy.” J. Microsc. 214 (1): 7–12.

Felsberg M, Sommer G. The monogenic signal. IEEE Transactions on Signal Processing 49, 3136-3144 (2001).

Larkin, KG, DJ Bone, and MA Oldfield. 2001. “Natural Demodulation of Two-Dimensional Fringe Patterns. I. General Background of the Spiral Phase Quadrature Transform.” Journal of the Optical Society of America A 18 (8): 1862–70.

Shribak, Michael, Kieran G. Larkin, and David Biggs. 2017. “Mapping Optical Path Length and Image Enhancement Using Quantitative Orientation-Independent Differential Interference Contrast Microscopy.” Journal of Biomedical Optics 22 (1): 016006–016006. doi:10.1117/1.JBO.22.1.016006.

Unser, M., D. Sage, and D. Van De Ville. 2009. “Multiresolution Monogenic Signal Analysis Using the Riesz #x2013;Laplace Wavelet Transform.” IEEE Transactions on Image Processing 18 (11): 2402–18. doi:10.1109/TIP.2009.2027628.

Reviewer #3 (Remarks to the Author):

Review:
From the point of view of DIC image handling for visualization and conversion of DIC –imaged structures into “bright objects” the proposed scheme is understandable. However, it seems partly empirically designed as a deeper foundation of the presented approach in signal/image processing is missing.
There should theoretical foundations and connections, as well as discussion of local orientation estimation be included like given in for example in:
• M. Unser et al.: Riesz transform and steerable wavelet frames
• S. Bernstein et al. Generalized Analytic Signals in Image Processing: Comparison, Theory and Applications

The theoretical background and novelty to existing reconstruction approaches based on Riesz Transform and/or Hilbert transform is not completely reviewed. This part should particularly be improved and extended.
Discuss novelty, similarities, and differences of presented approach to publications like:
• K.G. Larkin, P. A: Fletcher; Biomed Opt Express. 2014
• Shribak; Larkin; Biggs: Biomed. Opt. 22(1), 2017
• Z. Püspöki et al.; Transforms and Operators for Directional BioImaging Analysis; 2016

Point-by-point response to the comments for NCOMMS-17-11394

Reviewer 1

[General Comment #1]

In this paper, the authors present new applications of existing image analysis and signal processing techniques. In particular, they apply these techniques to determine whether cells display chiral biases. I appreciate the topic and the goal of the paper. <u>*However, the article is difficult to follow and the figure are not so convincing. Furthermore, the number of movies is overwhelming and they are not helpful. I would urge the authors to make their study more readable and to display the results in a clearer and more intuitive fashion.*</u>

[Answer to General Comment #1]

We are thankful to the reviewer for constructive comments on the manuscript. As depicted by the reviewer, our original manuscript was somewhat weak in clear description and data presentation. Following the suggestions by the reviewer, we have made the manuscript clearer and more readable in this revision. The major improved points are as follows:

1. Statistical data presentation might be confusing as pointed out by the reviewer. We used two levels of data; "individual" data and "summary" data. Individual data were collected from large samples (mostly from a large number of pixels or voxels) that were obtained in individual experimental trials (cells or cultures). Because these data do not follow a normal distribution, their bias from zero values was statistically tested by the non-parametric Wilcoxon signed-rank test. Summary data were composed of the mean values of the individual data. Their sample number is equal to the number of experimental trials. Because the summary data follow a normal distribution as predicted by "The Central Limit Theorem", their bias from zero was tested by the parametric one-sample t-test. To avoid a confusion and misunderstanding of readers, we have clearly stated the explanation of the two categories and the ways of statistical testing in the revised manuscript. In addition, we have presented the summary data only in the bar graphs of the main figures. All of the individual data were moved to Supplementary Information (data statistics to

Supplementary Table 2-5, histograms to **Supplementary Figs. 5-7**), except for the data corresponding to the pictures (numbers in **Fig. 4b-e**, and **Fig. 5f** graph corresponding to **Fig.5e** picture).

2. We have reduced the number of movies from 20 to 12, as suggested by the reviewer. Movies that are not essential were removed. Movies representing different views of the same samples were presented side-by-side in single movies to facilitate the understanding of the readers.
3. We have reduced the number of colors from the pictures. We used a combination of red/blue/gray instead of red/blue/green (**Fig. 4 and 7**). We have also reduced unnecessary colors from the drawings as many as possible.
4. As depicted by the reviewer, it seems difficult for readers to conceptually understand the relationship between the chiral phenomena described in separate sections of the original manuscript. In the revised manuscript, we have added a new figure (**Fig. 8**) that explains a schematic model on the relationship between a series of chiral behaviors with description of previously-known, newly-found and presumptive mechanistic insights. This figure will facilitate the understanding of the readers.

[Specific Comment #1-1]

Page 8: "By visual observation, individual filopodia not only twirled in the direction of a right-handed screw as previously reported but also appeared to exhibit axial spinning. They seemed to show retrograde motion toward the growth cone body, consistent with the retrograde actin flow."

I simply don't see this from the figure or the movies.

[Answer to Specific Comment #1-1]

As mentioned by the reviewer, it might be difficult for the readers to visually percept 3D motion from these movies. Thus, we have performed particle tracking analysis by tracking of voxels within the growth cone along the time axis. The results are shown as **Supplementary Movie 7**, which visualizes 3D helical motion of particles. The original sentences were moved to page 10 of the revised manuscript, and were rewritten as follows:

Page 10, Line 227:

"Particle tracking analysis (**Supplementary Movie 7**) further visualized that individual filopodia not only twirled in the direction of a right-handed screw as previously reported[6] but also exhibited axial spinning. They also showed retrograde motion toward the growth cone body, consistent with the retrograde actin flow[38]."

[Specific Comment #1-2]

Fig. 4d is supposed to show us that right-screw rotation is dominant over left-screw. I don't quite see that; there are too many colors in the figure.

[Answer to Specific Comment #1-2]

As mentioned in Answer to General Comment #1, we removed green color from the pictures by replacing it with gray color. We also added a picture showing RT-DIC intensity alone with gray color in **Fig. 4a**, and a picture showing velocity magnitude in **Fig. 4b**. Blue color (retraction) dominance in **Fig. 4d** and red color (right-screw) dominance in **Fig. 4e** will be easily recognized in the new pictures.

[Specific Comment #1-3]

Also, the quantification of this is in Fig 4g.

Presumably, the values next to the distribution represent the average and SD. The average is slightly positive which, I guess, means right-screw dominance. This is not explained. Furthermore, the average is very close to zero, can we really draw any conclusions from this? In other words, do we know that this distribution is skewed in a statistically significant way?

[Answer to Specific Comment #1-3]

We thank the reviewer for pointing out our failure. We forgot to explain the meaning of the values (mean ± s.d) in **Fig. 4e-g** of the original manuscript. In the revised manuscript, these histograms were moved to **Supplementary Fig. 5a-c** with the legends stating the meaning of the values. The values in the old **Fig. 4e-g** were also shown in the bottom of the new **Fig. 4b-d**.

Statistics of all “individual” data including those of the example (cell #2) shown in the histograms were listed in **Supplementary Table 2**. All axial data were statistically tested for the null hypothesis that the median value is equal to zero by the non-parametric Wilcoxon signed-rank test (**Supplementary Table 2**). The results indicate that almost all of the individual axial data (both axial velocity and axial angular velocity data) are significantly biased from zero with low P-values ($P<0.0001$).

[Specific Comment #1-4]

Why are the numbers of Fig. 4e and table 4i different? Do they represent different velocity magnitudes?

[Answer to Specific Comment #1-4]

The number in **Fig. 4e** of the original manuscript represents the value (mean ± s.d.) among all voxels in all frames ($n=2.17 \times 10^7$ voxels) of cell #2, which is shown in the pictures of **Fig. 4b,d**. This is an example of “individual” data. On the other hand, the numbers in **Fig. 4i** show the “summary” data that represent the mean ± s.e.m. of the mean of the individual cell data (n=8 cells). In the revised manuscript, the numbers at the bottom of the picture **Fig. 4b,d,e** represent the individual data of cell #2 (same as the numbers in the old **Fig. 4e-g**), while the graphs in the new **Fig. 4f** represent the summary data from 8 cells. We have rewritten the legends of **Fig. 4** so that these differences are clearly understood.

[Specific Comment #1-5]

Fig. 5: the velocity distribution is more skewed than the one in Fig. 4 but, again, how do we know that this skewness is statistically significant?

[Answer to Specific Comment #1-5]

We thank the reviewer for pointing out the lack of data on the statistical test. We added the result of the statistical test in **Fig. 5f** and the legends of the revised manuscript. We found a significant bias from zero values in the signed curvature of this sample (**Fig. 5f**).

Fig. 5 shows a neurite growth pattern in still images taken from an aldehyde-fixed culture sample. We only analyzed the curvature, but not the velocity or angular velocity. Thus, we do not think that the direct comparison of the skewness among different physical quantities have meanings.
Reading the original manuscript again, we noticed that there was a logical gap between the explanation of 3D helical motion of growth cones in **Fig. 4** and that of 2D clockwise neurite growth in **Fig. 5**, which could give misleading interpretations to the readers on the relationship between these phenomena. Thus, we rewrote the sentences at the top of the section of 2D neurite growth as follows:

Page 11, Line 240:
"**Quantification of chiral pattern in clockwise neurite growth**
RT-DIC microscopy and the associated methods can be used to automatically analyze chiral patterns in various scales, dimensions and cell types. In the scale larger than the growth cone, the neurons extend neurites clockwise on 2D culture substrates[5, 6] (**Fig. 5a**). This 2D clockwise neurite growth is driven by the 3D right-screw rotation of the growth cone filopodia[6], which is newly found to be accompanied by the 3D helical motion as described in the previous section."

We further presented a schematic model in **Fig.8** of the revised manuscript. This scheme will help the readers to conceptually understand the hierarchical relationship of chiral behaviors in the neurons.

[Specific Comment #1-6]
Fig. 6: Please show a quantitative analysis of the cell trajectories, including the biased clockwise motion.

[Answer to Specific Comment #1-6]
We thank the reviewer for the constructive suggestion that enhances the value of our work. In response to the reviewer's request, we have performed new experiment on the cell tracking analysis. The results are shown as new data and new movies (**Fig. 6d-g, Supplementary Fig. 6d-f, Supplementary Table 4 and Supplementary Movie 9,10**).

We have succeeded in tracking of individual cells moving in 10 mm^2 areas for 9 hours. Visual inspection of the movies, the cumulative angular displacements (**Fig. 6f**) and the statistics of the physical quantities (**Fig. 6g**) clearly and significantly show the clockwise bias. The newly-measured cell-wise quantities are comparable to the previously-measured pixel-wise values.

[Specific Comment #1-7]

<u>Also, how do cell-cell collision affect this bias?</u>

[Answer to Specific Comment #1-7]

To answer this question, we separately calculated the physical quantities in the presence or absence of cell-cell contacts that were judged from the distance of the cell centroids (**Supplementary Table 2** for individual data and **Fig. 6g** for summary data). We found that the mean values did not differ between the two states, but there was an increase in the variance of the quantities in the contact state. Accordingly, the curvature in the contact state did not show significant bias from zero. These data indicate that the clockwise bias is an intrinsic cell property and that this bias is perturbed by cell-cell contacts. Observation of the movies also revealed that rotational motion of the cells was independent of each other, suggesting that the bias is a cell-autonomous property.

[Specific Comment #1-8]

<u>Furthermore, how is the non-zero rotational motion along an axis perpendicular to the surface going to result curved trajectories? A more mechanistic insight will be helpful.</u>

[Answer to Specific Comment #1-8]

We thank the reviewer for raising the intriguing question. Rotational motion does not always generate a curved trajectory. If the rotation is composed of pure spin around the center of the cell without revolution, the cell center would move straight but each pixel would show cycloid-like complex motion. On the other hand, if the rotation is composed of pure revolution without spin, all pixels including the cell center would form circular trajectories. It is difficult to discriminate between the spin and the

revolution from pixel-wise data alone without their integration into cell-wise data. Thus, as suggested by the reviewer, cell-wise tracking data would become of particular importance for detection of the circular trajectories. The cell-wise data obtained in additional experiments (**Fig. 6g**) actually showed the presence of circular trajectories and a clockwise bias with comparable amount of quantities as similar as the pixel-wise data (**Fig. 6c**). These results support the presence of some mechanistic factors for generation of the revolution, but not those for the spin. Further mechanistic insights with relation to 3D rotational motion are discussed below in Answer #1-11 and **Fig. 8** of the revised manuscript.

[Specific Comment #1-9]

Fig. 7: This seems to show that a Dictyostelium cell has many tips. Normally, in 2D motion, it is believed that these cells have only 1-3 pseudopods. See, for example, Bosgraaf and van Haastert, Plos One, 2009. Please comment.

[Answer to Specific Comment #1-9]

In the analysis of the growth cones, we focused on the filopodia, but not on the lamellipodia. Analogically, in the analysis of *Dictyostelium* cells, we did not focused on the pseudopods but rather on the finer filopodial processes. Because the protrusions drawn in **Fig. 7d** of the original manuscript were ambiguous in shape, we replaced them with finer structures that look like filopodia. The diameter of interest can be controlled by the setting of the outer scale of the structure tensor. In the data shown in **Fig. 7**, we set the scale to the size comparable to the diameter of the filopodia. In preliminary experiments, we also analyzed the motility of the larger scale tip structure that may correspond to pseudopodia, but we could not detect a biased motility in such larger structures. In the revised manuscript, we added the following description with citation of Bosgraaf and Haastert (2009):

Page 13, Line 305:

“In this study, we focused on fine protrusions including filopodia, but not on pseudopodia[42], by setting the outer scale of the tensor, which is the diameter of interest, to about 0.6 μm.”

[Specific Comment #1-10]

Also, how is a tip defined and how is the radial axis defined?

[Answer to Specific Comment #1-10]

As mentioned above, we calculated the structure tensor focused on the structures with specific size (of diameter) at every voxel of 3D images. See **Supplementary Note 2** for detailed protocols. Then we calculate the tip certainty *Ct* for every voxel from the anti-parallel relationship between the eigenvector **e3** and polarity vector **ep** (the equation was described in the new scheme of **Fig. 7b**). Tip structures were extracted as the voxels with the certainty larger than the fixed threshold.

For the radial axis, we first calculated the intensity-weighted centroid that represents the center of the cell. The radial axis at the point of interest can be uniquely defined as the direction of the line connecting from the centroid to the point of interest (**Fig. 7b**).

[Specific Comment #1-11]

I also have a conceptual difficulty with this figure. It seems to show a right-screw rotation around the axis of motility. Fig. 6 seems to show a right-screw rotation perpendicular to the axis of motility. How are these two related?

[Answer to Specific Comment #1-11]

We thank the reviewer for depicting an important point. As the reviewer mentioned, the 90 degree conversion of the axis of rotation should be achieved. In the new figure (**Fig. 8**), we explain a schematic model explaining the conversion from the molecular chirality through 3D helical filopodial motility to 2D clockwise cell motility. See details in **Fig. 8** and the legends. Currently, we do not know the detailed molecular mechanisms of the conversion in *Dictyostelium* cells, but they can be predicted by the analogy with the findings in the neuronal growth cones. The explanation is as follows. In 2D environments, the filopodia attached to the substrate would not show rotational motion due to physical restriction, but those once detached from the substrate would freely rotate in the right-screw direction within the half hemisphere and attach to the

substrate again on the right side. Such motion would generate a rightward bias in the location of the attached filopodia. This asymmetry would cause rightward traction of the cell body, generating clockwise cell migration.

[Specific Comment #1-12]

Ref. 45 is not relevant to the clockwise or anti-clockwise motion of Dictyostelium aggregates. A better one is Rappel et al, Phys. Rev. Lett. 1999.

[Answer to Specific Comment #1-12]

We thank the reviewer for the comment. The reference was replaced with the suggested one in the revised manuscript.

Reviewer 2

[General Comment #2]

The combination and adaptation of methods from multiple disciplines is impressive and well described, and offers an insight into the origins and mechanisms of left-right asymmetry. A key advance in this manuscript is the introduction of a composite RT which enables the use of single-shot DIC capture to collect and subsequently analyse structural information. Single-shot methods are highly valuable for rapid capture of living 3D cells. I think the paper has the potential to be highly influential in demonstrating the application of advanced microscopy and image processing methods to a compelling biomedical problem.

[Answer to General Comment #2]

We are thankful to the reviewer for positive comments that correctly evaluate our work. We also thank for constructive comments that improve the value of the manuscript. As depicted by the reviewer, one of the most significant points of the paper lies in the development of a single-shot method with the composite RT for rapid DIC imaging. This method will complement the more precise multi-shot methods. In this revision, we have described the historical backgrounds of DIC imaging and Riesz transform and have emphasized the similarity and the novelty of our new methods with relation to the other methods, with citing the literature raised by the reviewer.

[Specific Comment #2-1]

The authors are careful to introduce the topics of image processing of DIC images and, separately, the Riesz transform. However, there is a significant history of application of the Riesz transform specifically to DIC images, which is currently not referenced at all:

• *Applying the inverse Riesz transform to images captured using an optical variant of DIC was first proposed in (Arnison, 2004) where it was used to integrate the phase gradient to estimate the linear phase.*

• *A recent paper by (Shribak, 2017) proposes using the inverse Riesz transform for visualisation of images captured using another optical variant of DIC. This paper also serves as a brief review of the last 13 years of literature on Riesz transforms and DIC.*

Although I have listed papers which use the inverse RT, the forward and inverse RT operations are closely related.

[Answer to Specific Comment #2-1]

In Introduction, we briefly reviewed the background of the RT and RT-based DIC imaging with citing all of the papers raised by the reviewer. The modified description is shown below:

<u>Page 4, Line 68:</u>

"To overcome this problem, many methods have been developed to date(Shribak, Larkin et al. 2017). One of the most efficient and convenient methods adopts acquisition of multiple phase gradient images with orthogonal shears and their integration by Riesz transform (RT)(Arnison, Larkin et al. 2004, Larkin and Fletcher 2014, Shribak, Larkin et al. 2017). RT(Felsberg and Sommer 2001), which was independently and simultaneously proposed as the spiral phase transform(Larkin, Bone et al. 2001), is a multidimensional extension of the 1D Hilbert transform (HT), and has recently been used in many fields of image processing and analysis(Unser, Sage et al. 2009, Unser and Van De Ville 2009, Bernstein, Bouchot et al. 2013, Püspöki, Storath et al. 2016). These restoration methods with multiple DIC images precisely restore original images, but they require special equipment and multi-shot image acquisition that is disadvantageous for fast 3D live imaging. A method for single-shot DIC imaging with HT was also developed(Arnison, Cogswell et al. 2000), but it cannot detect objects along the shear direction. Here, we developed a simple but efficient method for single-shot DIC images with a composite Fourier filtering based on the directional RT(Unser, Sage et al. 2009). This composite RT, utilizing both phase gradient and absorption information of DIC images, converts a shadow-cast DIC image into a self-luminous intensity image. This improved DIC microscopy with the composite RT, called RT-DIC microscopy, was applied to 3D time-lapse imaging of photosensitive structures."

[Specific Comment #2-2]

These papers all rely on multi-shot variants of DIC, so the use of the RT with single-shot DIC in the manuscript remains novel. The claims on page 14 that the proposed method can be used with standard a DIC microscope are justified.

[Answer to Specific Comment #2-2]

We thank the reviewer for positive comments pointing the novelty of our work. In the revised manuscript, we emphasized this point by adding the following sentences.

Page 15, Line 334:

Although RT-DIC microscopy with single-shot imaging is not suitable for complete restoration of shear-independent rotation-invariant images like the techniques with multi-shot imaging(Arnison, Larkin et al. 2004, Larkin and Fletcher 2014, Shribak, Larkin et al. 2017), it is highly advantageous for rapid capture of 3D live images. In addition, RT-DIC microscopy uses a standard conventional DIC microscope and does not require any additional special equipment.

[Specific Comment #2-3]

An additional issue is that while the manuscript correctly cites (Felsberg, 2001), the Riesz transform as a 2D extension of the 1D Hilbert transform was independently and simultaneously proposed by (Larkin, 2001). The manuscript specifically relies on a directional RT, which was introduced by (Unser, 2009).

[Answer to Specific Comment #2-3]

We correctly cited the papers (Larkin, 2001; Unser, 2009) in the revised manuscript. The modified description in *Introduction* was previously mentioned in Answer #2-1. We also cited these works in *Results* (Page 6, Line 114-116) and **Supplementary Note 1** (Page 17, Line 299 and Page 18, Line 305).

[Specific Comment #2-4]

Given the importance of the RT to the methods of the paper, and the weight being placed on the novelty of the RT being used in this context, I would recommend revisions

be made to clarify the relationship between the composite RT method proposed in the manuscript, and the literature on RT and DIC.

[Answer to Specific Comment #2-4]

As mentioned in Answers #2-1, #2-2 and #2-3, we briefly reviewed the history of RT and DIC with citing the literature raised by the reviewer, and described the similarity and novelty of the single-shot methods with the composite RT. We show the list of modification again below:

Introduction	Page 4, Line 68-83
Results	Page 6, Line 114-116
Discussion	Page 15, Line 334-337
Supplementary Note 1	Page 17, Line 299 – Page 18, Line 305

[Minor Comment #2-1]

1. Page 19, Line 402: "the intensity was expanded to a full 12-bit range." What does expanded mean? The camera captured data at 12-bit, so working in 12-bits during processing is merely maintaining, not expanding, the range. In addition, I would expect that the Fourier processing described would likely be performed in MATLAB in single precision float (32-bit).

[Answer to Minor Comment #2-1]

In "expansion", the maximum and minimum intensity was calculated from the source DIC images. Then, the intensity was proportionally adjusted so that the maximum is reset to 4095 and the minimum is reset to zero. As pointed out by the reviewer, this process does not change the quality of the DIC image or affect the subsequent image processing. We have many sets of image data taken with various gains and offsets. We used "expansion" for the purpose of visualization of the source DIC image with the same condition. This normalization is especially useful for overlay of the processed RT-DIC image on the source DIC image without adjusting the intensity level of the DIC image sample-by-sample.

Single precision float would be enough for calculation of Fourier transform, but we actually use double precision float throughout the processing to suppress the numerical

errors. Double precision float is required for treating a large number of voxel data (~1×10^9 points) whose values range from extremely small numbers to extremely large numbers.

[Minor Comment #2-2]

2. Page 20, lines 413-414. The images were "often" deconvolved. Please clarify "often", as this is overly vague. Deconvolution requires a calibrated or estimated PSF. How was the PSF determined?

[Answer to Minor Comment #2-2]

We thank the reviewer for the comment pointing out our error. We forgot to change the description in the premature version of the manuscript. Deconvolution was applied to "all" 3D images. We used a PSF determined by the open source software "PSF Generator". The description was corrected as follows:

Page 21, Line 456:

"All 3D images were deconvoluted with open-source software DeconvolutionLab2(Sage, Donati et al. 2017) with theoretical point spread function (PSF) based on Born-Wolf model generated by PSF Generator(Kirshner, Aguet et al. 2013)."

Reviewer 3

[General Comment #3-1]

From the point of view of DIC image handling for visualization and conversion of DIC –imaged structures into "bright objects" the proposed scheme is understandable. However, it seems partly empirically designed as a deeper foundation of the presented approach in signal/image processing is missing.

There should theoretical foundations and connections, as well as discussion of local orientation estimation be included like given in for example in:

• M. Unser et al.: Riesz transform and steerable wavelet frames

• S. Bernstein et al. Generalized Analytic Signals in Image Processing: Comparison, Theory and Applications

[Answer to General Comment #3-1]

We thank the reviewer for the valuable comments for improvement of the paper. According to the reviewer's suggestion, in the revised version, we described and reviewed the theoretical foundations on the Riesz transform and its applications with citing these articles. The sections of Introduction, Results, Discussion and Supplementary Notes were modified as follows in the revised manuscript:

Introduction: Page 4, Line 68:

"To overcome this problem, many methods have been developed to date(Shribak, Larkin et al. 2017). One of the most efficient and convenient methods adopts acquisition of multiple phase gradient images with orthogonal shears and their integration by Riesz transform (RT)(Arnison, Larkin et al. 2004, Larkin and Fletcher 2014, Shribak, Larkin et al. 2017). RT(Felsberg and Sommer 2001), which was independently and simultaneously proposed as the spiral phase transform(Larkin, Bone et al. 2001), is a multidimensional extension of the 1D Hilbert transform (HT), and has recently been used in many fields of image processing and analysis(Unser, Sage et al. 2009, Unser and Van De Ville 2009, Bernstein, Bouchot et al. 2013, Püspöki, Storath et al. 2016). These restoration methods with multiple DIC images precisely restore original images, but they require special equipment and multi-shot image acquisition that is disadvantageous for fast 3D live imaging. A method for single-shot DIC imaging with

HT was also developed(Arnison, Cogswell et al. 2000), but it cannot detect objects along the shear direction. Here, we developed a simple but efficient method for single-shot DIC images with a composite Fourier filtering based on the directional RT(Unser, Sage et al. 2009). This composite RT, utilizing both phase gradient and absorption information of DIC images, converts a shadow-cast DIC image into a self-luminous intensity image. This improved DIC microscopy with the composite RT, called RT-DIC microscopy, was applied to 3D time-lapse imaging of photosensitive structures."

<u>Results: Page 6, Line 114-116</u>

RT(Felsberg and Sommer 2001, Larkin, Bone et al. 2001) is the multidimensional extension of Hilbert transform (HT), which shifts the phase of 1D signals by 90° (Supplementary Note 1). We utilized a directional form of RT(Unser, Sage et al. 2009).

<u>Supplementary Note 1: Page 17, Line 299 – Page 18, Line 305</u>

The Riesz transform (RT), an extension of the HT to multi-dimensions, is used for generation of the multidimensional analytic signal, the monogenic signal(Felsberg and Sommer 2001). The RT is also independently and simultaneously proposed as the spiral phase transform(Larkin, Bone et al. 2001).

The directional RT(Unser, Sage et al. 2009) toward n can be defined by a multiplier:

[General Comment #3-2]

<u>*The theoretical background and novelty to existing reconstruction approaches based on Riesz Transform and/or Hilbert transform is not completely reviewed. This part should particularly be improved and extended.*</u>

<u>*Discuss novelty, similarities, and differences of presented approach to publications like:*</u>

- *K.G. Larkin, P. A: Fletcher; Biomed Opt Express. 2014*
- *Shribak; Larkin; Biggs: Biomed. Opt. 22(1), 2017*
- *Z. Püspöki et al.; Transforms and Operators for Directional BioImaging Analysis; 2016*

[Answer to General Comment #3-2]

Following the reviewer's suggestion, we modified the description in the revised manuscript. The modified points are included in the texts of Answer #3-2. In addition, we modified the following points in *Discussion*:

<u>Page 15, Line 334:</u>

Although RT-DIC microscopy with single-shot imaging is not suitable for complete restoration of shear-independent rotation-invariant images like the techniques with multi-shot imaging(Arnison, Larkin et al. 2004, Larkin and Fletcher 2014, Shribak, Larkin et al. 2017), it is highly advantageous for rapid capture of 3D live images. In addition, RT-DIC microscopy uses a standard conventional DIC microscope and does not require any additional special equipment.

<u>Reviewers' Comments:</u>

Reviewer #1 (Remarks to the Author):

The authors have substantially rewritten their manuscript. In addition, they have performed additional and clarifying experiments. I think the paper is now suitable for publication in the journal.

Reviewer #2 (Remarks to the Author):

I am satisfied that the author's revisions address the points raised in my review.

Reviewer #3 (Remarks to the Author):

With respect to the additional information which has been given
now the paper is better to understand. Aswell the references has been included properly.
I would suggest only for your personal interest to quantify the quantitativeness of your single DIC image method of reconstruction for a synthetic test image, e.g. for a blurred circular phase object, in comparison with a two or multi-DIC image reconstruction approach.

Point-by-point response to the comments for NCOMMS-17-11394B

Reviewer 3

[Comment #1]

With respect to the additional information which has been given now the paper is better to understand. Aswell the references has been included properly.

I would suggest only for your personal interest to quantify the quantitativeness of your single DIC image method of reconstruction for a synthetic test image, e.g. for a blurred circular phase object, in comparison with a two or multi-DIC image reconstruction approach.

[Answer to Comment #1]

Following the suggestion by the reviewer, we added a new figure (**Supplementary Fig. 1**) that compares the conversion efficiency of our single-shot method using the composite Riesz transform with that of the previous multi-shot method using the inverse Riesz transform. An explanation of this figure was also added at the end of the first section of *Results*. The data show that our single-shot method is not superior to the multi-shot method, but has comparable efficiency enough for practical use.
